# Infection by High-Risk Human Papillomaviruses, Epithelial-to-Mesenchymal Transition and Squamous Pre-Malignant or Malignant Lesions of the Uterine Cervix: A Series of Chained Events?

**DOI:** 10.3390/ijms222413543

**Published:** 2021-12-17

**Authors:** Giovanni Barillari, Roberto Bei, Vittorio Manzari, Andrea Modesti

**Affiliations:** Department of Clinical Sciences and Translational Medicine, University of Rome Tor Vergata, 1 via Montellier, 00133 Rome, Italy; bei@med.uniroma2.it (R.B.); manzari@med.uniroma2.it (V.M.); modesti@med.uniroma2.it (A.M.)

**Keywords:** HPV, inflammation, p53, hypoxia, EMT, uterine SIL, cancer stem cells, uterine cervical carcinoma

## Abstract

Wound healing requires static epithelial cells to gradually assume a mobile phenotype through a multi-step process termed epithelial-to-mesenchymal transition (EMT). Although it is inherently transient and reversible, EMT perdures and is abnormally activated when the epithelium is chronically exposed to pathogens: this event deeply alters the tissue and eventually contributes to the development of diseases. Among the many of them is uterine cervical squamous cell carcinoma (SCC), the most frequent malignancy of the female genital system. SCC, whose onset is associated with the persistent infection of the uterine cervix by high-risk human papillomaviruses (HR-HPVs), often relapses and/or metastasizes, being resistant to conventional chemo- or radiotherapy. Given that these fearsome clinical features may stem, at least in part, from the exacerbated and long-lasting EMT occurring in the HPV-infected cervix; here we have reviewed published studies concerning the impact that HPV oncoproteins, cellular tumor suppressors, regulators of gene expression, inflammatory cytokines or growth factors, and the interactions among these effectors have on EMT induction and cervical carcinogenesis. It is predictable and desirable that a broader comprehension of the role that EMT inducers play in SCC pathogenesis will provide indications to flourish new strategies directed against this aggressive tumor.

## 1. Introduction

Epithelial cells lining human organs are tightly joined together by means of adherens junctions, are connected to the extracellular matrix via membrane receptors, are oriented according to an apical–basal polarity, and have a limited life span [1].

In multilayered epithelia, dead cells are replaced by young ones arising from the differentiation of stem cells located in the basal layers [1]. This turnover is altered during the repair of a damaged epithelium when epithelial cells died due to the action of harmful agents are replaced in part by the differentiation of stem cells, and in part by the proliferation and migration of epithelial cells that are close to the site of damage [1,2]. Specifically, cells that have survived the harm proliferate due to the ending of contact inhibition and, at the same time, migrate to the site of damage [1,2]. For this to happen, epithelial cells change their phenotype from static to mobile through the EMT process [1,3]. The latter entails sequential events leading epithelial cells to gradually assume a mesenchymal phenotype [1,3]. This being so, in tissues undergoing development, remodeling, or repair, cells that are in diverse states of differentiation, intermediate between the fully epithelial and the fully mesenchymal phenotype, can be simultaneously present [4,5].

The repair process of an epithelium results from an initial inflammatory phase, a midst proliferative phase, and a final, tissue remodeling phase [2,6]. Ultimately, EMT is triggered by inflammatory mediators and sustained by growth, chemotactic, or differentiation factors [1,3]. In epithelial cells, most of these molecules spark signaling pathways which, in turn, activate transcription factors including zinc finger E-box-binding homeobox (Zeb) 1 or 2, basic helix-loop-helix twist homolog (Twist) 1 or 2, and zinc finger SNAI 1 (Snail) or 2 (Slug) proteins [7,8,9,10,11].

The above-mentioned transcriptional regulators act together with noncoding RNAs and chromatin or histone modifiers at promoting the expression of mesenchymal markers while repressing that of the epithelial ones [12,13,14,15,16]. Consequently, epithelial cells which have undergone EMT display a reduced expression of epithelial adherens junction components, such as the epithelial (E)-cadherin, that are replaced with mesenchymal adhesion molecules, like neuronal (N)-cadherin [3]. Other changes in intercellular adhesiveness involve the upregulation of claudin-1 [17], a tight junction molecule that mediates epithelial cell invasion and migration [18]. Moreover, molecules of the epithelial cytoskeleton, such as the cytokeratins, are replaced by components of the mesenchymal cytoskeleton (e.g., vimentin): accordingly, the shape of epithelial cells is converted from cobblestone-like (typical of static epithelial cells) to spindle-like (characteristic of the highly mobile mesenchymal cells) [3,19]. In the meantime, trans-differentiated epithelial cells synthesize enzymes actively digesting the interstitial or pericellular matrices [20].

Variations in the expression of intercellular adhesion or cytoskeletal molecules, as well as the proteolytic degradation of the matrix, cause epithelial cells to separate from each other, lose their apical–basal polarity, and acquire the migratory capabilities that render possible the repair of the damaged epithelium [3].

When the tissue is repaired, cells stop proliferating and moving, and their phenotype is reconverted from mesenchymal to epithelial, through a process termed mesenchymal-to-epithelial transition (MET): the latter involves molecular changes such as N-cadherin or vimentin replacement with E-cadherin and the keratins, respectively [21]. MET is induced by transcription factors (e.g., ELF3/5, GRHL2, and OVOL1/2) or noncoding regulatory RNAs which counteract the activity of the EMT-promoting transcriptional regulators [22,23,24,25,26,27,28,29,30,31,32].

Thus, the EMT that accompanies tissue repair is a transient process, which is very similar to the EMT that occurs during embryogenesis or body growth [3,21]. Precisely because they are transitory and reversible, these types of EMT are physiological [3,21].

However, when the epithelium is subjected to the prolonged action of detrimental agents, EMT persists, leading to severe tissue alterations which, in turn, may form the basis for the development of various pathologies [3,5,6,21].

In particular, EMT participates in the onset, progression, and metastatic spread of carcinomas. This is because EMT: (i) renders epithelial cells susceptible to malignant transformation [33,34]; (ii) facilitates the detachment of transformed epithelial cells from the primary tumor [5,35,36]; (iii) promotes epithelial cell invasion of the peri-tumor matrix and the basement membrane [36,37,38,39,40,41,42,43,44,45]; (iv) favors the locomotion of transformed epithelial cells and their spreading throughout the body [21,46,47,48,49]; (v) increases the survival of carcinoma cells that have detached from the primary tumor and have reached the circulatory bed or the new site of metastases [50,51,52]; (vi) reprograms the metabolism of carcinoma cells, adapting it to the changed characteristics of the new microenvironment [53,54,55,56].

Among EMT-linked epithelial malignancies is SCC of the uterine cervix, which is the most frequent cancer of the female genital system [57]. Although preventative screenings have significantly decreased cervical SCC-related deaths [58], this malignancy is still a major cause of mortality throughout the world because of its high rate of recurrence and/or metastasization [57]. It is now widely accepted that the aggressive clinical behavior of SCC is triggered by the abnormal and prolonged EMT occurring in its lesions [57].

In view of these findings, the present review deals with the mechanisms by which EMT is induced in uterine cervical SCC, favoring the clinical progression and metastasization of this tumor.

## 2. The E5, E6, and E7 Proteins of HR-HPVs Trigger EMT in Cervical Epithelial Cells

The cervix is the lower end of the uterus and consists of two different areas: the endocervix, which continues with the body of the uterus, and the ectocervix which projects into the vaginal cavity [59]. While the endocervix is lined with a monostratified cylindrical epithelium, the ectocervix is covered by a multilayered non-keratinized squamous epithelium that is continuously renewed via the migration of immature cells of the basal layers towards the superficial layers, where cells differentiate until they undergo desquamation [60,61,62].

During puberty, the cylindrical epithelium that covers the area of the endocervix next to the ectocervix is replaced by a multilayered squamous epithelium: this area, which is termed the “transformation zone”, is the one in which SCC develops most frequently [58,60,61,62].

The onset of SCC is for almost all cases associated with infection with DNA viruses belonging to the HPV family, with HPV16 being the most prevalent HR-HPV type [62,63,64].

Once sexually transmitted, HR-HPVs reach the cells of the basal layer of the ectocervical epithelium: there the viruses actively replicate, this implying the expression of the viral genome and the synthesis of its products, the E5, E6, and E7 transforming proteins included [62,63,64,65,66,67,68,69].

In the epithelial cells of the uterine cervix basal layer, E5 increases the protein levels of the receptor for the highly mitogenic epidermal growth factor (EGF) via a block of its degradation that normally follows its stimulation by EGF [68,69]. In addition, E5 enhances the mitogenic activity of endothelin-1 and downregulates the expression of the cyclin-dependent protein kinase inhibitors p21^WAF/CIP1^ and p27^KIP1^, thus promoting cell cycle progression [69]. Furthermore, E5 hampers the adhesive interactions among epithelial cells mediated by connexin 43: this reduces the contact inhibition, further contributing to the proliferation of epithelial cells [68]. At the same time, E5 inhibits epithelial cell differentiation by downregulating the expression of the fibroblast growth factor receptor (FGFR)2b [68,69,70]. Because of all these activities, E5 favors the proliferation of immature basal cells and, at the same time, hampers their differentiation (Table 1) [68,69,70].

The findings summarized herein are from references [68,69,70]. Abbreviations: AKT: protein kinase B; Bax: B-cell lymphoma 2-associated X protein; CD: cluster of differentiation; cx: connexin; EC: epithelial cells; EGFR: epidermal growth factor receptor; EMT: epithelial-to-mesenchymal transition; ET: endothelin; Fas: tumor necrosis factor receptor superfamily member 6; FGFR: fibroblast growth factor receptor; HPV: human papillomavirus; MAPK: mitogen-activated protein kinase; MHC: major histocompatibility complex.

**Figure 1:**
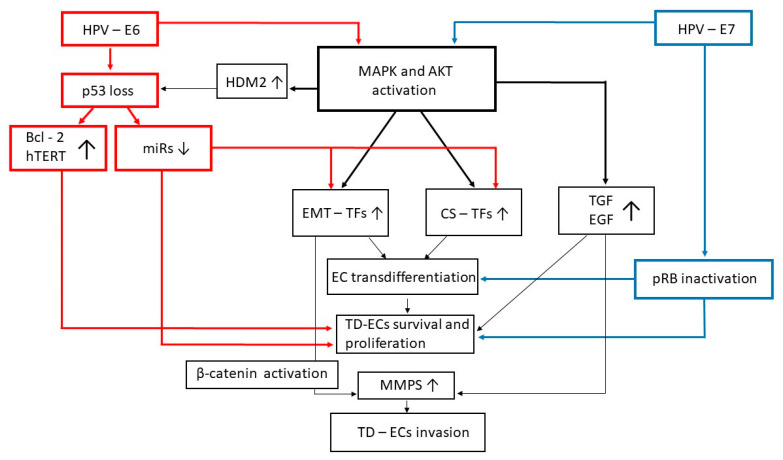
The E6 and E7 proteins of high-risk HPVs exert activities that make them capable of directly converting epithelial cells into mesenchymal, stem-like cells. Arrows symbolize directions of connections. Abbreviations: AKT: protein kinase B; CS: cellular stemness; EC: epithelial cells; EGF: epidermal growth factor; EMT: epithelial-to-mesenchymal transition; HPV: human papillomavirus; HDM2: human double minute 2; hTERT: human telomerase reverse transcriptase; MAPK: mitogen-activated protein kinase; miR: microRNA; MMP: matrix metalloproteinase; pRb: retinoblastoma protein; TD: trans-differentiated; TF: transcription factor; TGF: transforming growth factor.

For its part, the HPV-E6 protein drives the degradation of the p53 oncosuppressor cellular protein via the ubiquitin ligase-cellular proteasome system (Figure 1) [51,65]. This event is followed by the upregulation of p53-repressed factors, such as the Bcl-2 protein and the telomerase enzyme (Figure 1), leading to an alteration in the kinetic of cervical epithelial cell renewal [50,51,66].

Simultaneously with the effects promoted by E5 and E6, the HPV-E7 protein binds and inactivates the retinoblastoma tumor suppressor cellular protein (pRb) (Figure 1), thus synergizing with E5 and E6 in impeding infected cells to exit the cell cycle and differentiate [66].

It is noteworthy that, while proliferating, HPV-infected basal epithelial cells migrate towards the superficial layers of the cervical epithelium [62]. In the majority of cases, these HPV-promoted phenomena are limited to causing a thickening of uterine ectocervical epithelium or, at most, benign flat warts [62]. In a small percentage of cases, however, the abnormal growth of immature basal cells and their migration towards the superficial layers of the cervical epithelium lead to the development of hyperplastic and/or dysplastic lesions termed squamous intraepithelial lesions (SILs) [62,71,72].

Two types of SILs are known: low-grade SIL (L-SIL) and high-grade SIL (H-SIL) [61,71,72].

L-SIL, also defined as grade 1 cervical intraepithelial neoplasia, consists of proliferating and keratinized immature basal cells whose nuclei are surrounded by vacuoles: these cells represent the manifestation of a productive HPV infection and can constitute up to 1/3 of the cervical epithelium [71,72]. L-SIL generally undergoes spontaneous involution until it disappears, progressing into H-SIL only in 20–30% of cases [71,72,73].

H-SIL encompasses grade 2 and 3 cervical intraepithelial neoplasia. The former is characterized by hyper-keratinized epithelial cells that colonize 2/3 of cervical basal layers [71,72]; whereas grade 3 cervical intraepithelial neoplasia represents the early stages of HPV-induced carcinogenesis, and it is made up of highly dysplastic cells that undergo atypical mitosis and occupy the 2/3 of the entire epithelium, including the superficial layers [71,72].

When its DNA does not integrate into the genome of the host cell, HPV is generally neutralized by the immune system: under this circumstance, the abnormal proliferation of basal cells ceases, the warts and SILs regress and the cervical epithelium returns to normal [62].

However, the E5, E6, and E7 oncoproteins actively counter host immune response directed against the HR-HPVs. Specifically, E5 reduces the levels of the CD1d receptor on the plasma membrane of HPV-infected epithelial cells, thereby impairing their recognition by natural killer cells (Table 1) [69]. In addition, E5 can retain the major histocompatibility complex/human leukocyte (MHC/HLA) class I antigens in the endoplasmic reticulum: as a consequence, E5-expressing epithelial cells display low MHC/HLA-I antigen levels on their surface (Table 1), and this jeopardizes their disruption by cytotoxic T cells [68,69]. In addition, E5 further hinders the clearance of HR-HPV-infected cervical epithelial cells by downregulating the expression of MHC class II antigens induced by the inflammatory mediator interferon (IFN)γ on the surface of these cells [68,69]. For their part, the E6 and E7 proteins of the HR-HPVs halt IFN capability of reverting the inhibitory effect that E5 has on MHC/HLA-I [68,69]. Moreover, E5 prolongs the survival of HR-HPV-infected epithelial cells by downregulating the expression of the cell death Fas receptors and by promoting the degradation of the proapoptotic Bax protein (Table 1) [68,69]. The inhibition of the anti-HPV immune response and, in general, the reduction in the apoptosis of cervical cells infected with these viruses, extends the duration and the intensity of HR-HPV infection, thus increasing the likelihood of cellular transformation. In fact, when E5, E6, and E7 succeed at hindering the immune response and HR-HPV load is high, the viral DNA integrates into the genome of host cells [74,75,76,77]: in such an eventuality the HPV-E5 gene is lost, while the HPV-E6 and E7 proteins are overexpressed and their carcinogenic effects are intensified [66,67,68,69,77]. As a consequence, the entire cervical epithelium is replaced by poorly differentiated cells displaying abnormal nuclei and atypical mitoses [71,72]. In 20–50% of H-SIL cases, these cells may degrade the epithelial basement membrane via the synthesis of proteolytic enzymes, thus initiating the development of an invasive SCC [73,78].

Of importance, results from clinical studies indicate that, as compared to normal cervical epithelium, the expression of the EMT-promoting Zeb 1, Twist 1 or 2, and Snail transcription factors is progressively increased during the onset of uterine cervical SIL and its progression to SCC [79,80,81,82,83,84,85,86]. Consequently, the level of both claudin-1 and vimentin increases, while that of E-cadherin is reduced [17,87]. These phenomena parallel L-SIL progression to H-SIL and finally to SCC, and are predictive of SCC metastasis to lymph nodes [17,87].

As for physiological EMT, that also accompanies SCC onset or progression occurs gradually, explaining why cells with intermediate phenotypes between the fully epithelial and the decidedly mesenchymal ones can be found in the squamous lesions of the uterine cervix [88,89,90,91,92,93,94,95,96,97]. Such a variety of phenotypes could depend on the multitude of EMT promoters that may be present in the cervix of HPV-infected women, and on the temporal sequence according to which cervical epithelial cells are exposed to these factors.

In this context, it has to be highlighted that, while downregulating the epithelial FGFR2b, HPV-E5 drives the expression of the mesenchymal FGFR2c variant, whose signaling leads to EMT, cellular invasiveness, and tumorigenic behavior (Table 1) [70].

Still in this regard, in vitro studies performed with SCC cell lines have shown that the E6 and E7 proteins of HR-HPVs can directly trigger EMT via the induction of Twist2, Zeb, Slug, or Snail expression and nuclear translocation [83,98,99,100]. This is because both the E6 and E7 proteins of HR-HPVs can turn on the mitogen-activated protein kinases (MAPK)/extracellular-regulated kinases (ERK) and/or the phosphoinositide 3 kinase (PI3K)/protein kinase B (AKT)/mammalian target of rapamycin (mTOR) pathways (Figure 1) [101,102,103,104]. Triggering of these signaling pathways actuate the expression of Snail, Slug, Twist, and Zeb, as well as other EMT-promoting transcription factors (Figure 1), including STAT3 and nuclear factor-κB (NF-κB) [7,8,9,10,11].

On its part, also E5 can turn on both PI3K/AKT and MAPK/ERK (Table 1), either directly or via EGFR activation [68,69]: this explains why in cervical L-SILs the expression of HPV-E5 parallels that of the Snail, Slug or Zeb transcription factors [70].

Moreover, E5, E6, and E7 enhance the activity, and/or upregulate the expression, of EMT promoters such as EGF and transforming growth factor (TGF)-β1 (Table 1 and Figure 1) [68,69,100,105].

Undoubtedly, however, the induction of EMT in HPV-infected cervical epithelial cells strongly depends on E6’s capability of driving p53 degradation [51]. In fact, this event causes p53 to no longer activate the transcription of microRNAs (miRs) that otherwise would have blocked Zeb, Slug, or Twist expression (Figure 1), thereby hindering EMT [34,106,107,108,109].

miR-34 and miR-203, together with the miR-200 family members, are among the p53-transcribed miRs which are reduced in HPV-infected epithelial cells: downregulation of these miRs follows E6-promoted p53 degradation, and it is paralleled by an increase in Snail or Zeb levels, EMT and cell invasion (Figure 1) [34,51,107]. Since the p53-transcribed miRs repress not only the phenotypic plasticity, but also the survival, growth, invasion, and migration of SCC cells, a reduction in their expression influences not only the phenotype of SCC cells but also the clinical course of SCC [106,110,111,112,113]. For instance, miR-34 levels are significantly lower in L-SIL than in normal cervical epithelium, even lower in H-SIL, and extremely low in invasive SCC [51]. Worthy of interest is the fact that given the pro-apoptosis effect and growth suppressive activities of miR-34, its downregulation in cervical SIL or SCC correlates with the increased survival and replicative capacity of HPV-infected cervical epithelial cells (Figure 1) [51].

These phenomena are amplified in cells where E6-induced p53 loss is accompanied by the functional impairment of pRB caused by the HPV-E7 oncoprotein. In fact, the simultaneous inactivation of p53 and pRB strongly upregulates Snail, Slug, and Zeb1 expression, and readily converts epithelial cells from static to motile and invasive (Figure 1) [114,115].

On the other hand, when overexpressed and/or hyperactivated, Snail, Slug, and Zeb1 can directly counteract p53 and pRb activity, further downregulating tumor suppressive miRs, and rendering epithelial cells susceptible to malignant transformation [33,34]. In agreement with these findings, the concurrent inactivation of p53 and pRB has been shown to give rise to mesenchymal-like tumors in animal models [116].

Taken together, all these findings indicate that the E5, E6, and E7 oncoproteins of the HR-HPVs cooperate in inducing the proliferation and trans-differentiation of cervical epithelial cells. However, it has to be highlighted that while in L-SIL lesions the DNA of HR-HPVs is found in the epithelial cytosol, most of HSILs and the very vast majority of SCCs are associated with viral DNA integration into the host cell genome [68,69,70]. In agreement with the fact that this event causes the loss of the E5 gene [68,69,77], the latter is expressed in cervical L-SILs [70] but not in cervical carcinomas [68]: this underlies the established belief that E5 plays a role mostly in the early stages of cervical carcinogenesis [63,66,67,68,69,77].

## 3. Inflammation Cooperates with the E6 and E7 Proteins of HR-HPVs at Promoting EMT in Normal or Neoplastic Uterine Cervical Epithelial Cells

**Figure 2:**
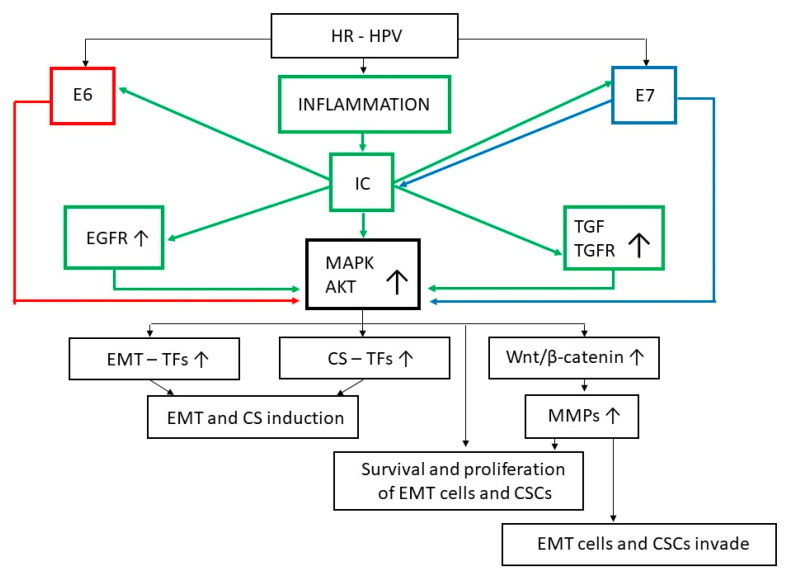
Inflammatory cytokines cooperate with the HPV-E6 and HPV-E7 proteins at inducing the appearance of epithelial/mesenchymal hybrids or stem-like cells, and at favoring their survival, growth, and invasiveness. Arrows symbolize directions of connections. Abbreviations: AKT: protein kinase B; CS: cellular stemness; CSC: cancer stem cell; EGFR: epidermal growth factor receptor; EMT: epithelial-to-mesenchymal transition; HR-HPV: high-risk human papillomavirus; IC: inflammatory cytokines; MAPK: mitogen-activated protein kinase; MMP: matrix metalloproteinase; TF: transcription factor; TGF: transforming growth factor; TGFR: transforming growth factor receptors; Wnt: wingless-type mouse mammary tumor virus integration site.

As with many other types of dysplastic or neoplastic lesions [117], the development of uterine cervical SILs and their progression to SCC are respectively preceded and accompanied by an inflammatory reaction [118,119,120,121]. The latter often follows the infection of the uterine cervix with HPV (Figure 2) as well as other microbial agents, and it accompanies the immune response directed against these pathogens and/or the reparation of the tissue damage caused by them [122,123].

Inflammation of the uterine cervix involves its infiltration by activated leukocytes releasing a variety of cytokines among which tumor necrosis factor (TNF)α, interleukin (IL)-1, or IL-6 are detected in most uterine SILs or SCCs [120,124,125,126,127]. Of interest, as with activated leukocytes, SCC cells also constitutively produce IL-1, IL-6, and TNFα [128,129,130], thereby contributing to an increase in the intratumoral concentration of these cytokines. In this context, it is significant that TNFα promotes the gene expression of HPV-E6 and HPV-E7, and that the latter protein upregulates TNFα expression in a reciprocal fashion (Figure 2) [131,132].

Noteworthy, IL-1, IL-6, or TNF tissue levels correlate positively with the stage of progression of cervical disease, from healthy epithelium to L-SIL, and from this to H-SIL and SCC [118,119,120,121,122,124,126,127,133].

At variance with IL-1, IL-6, or TNF, the concentrations of inflammatory IFNγ decrease in uterine cervical SILs or SCC, as compared to normal cervical epithelium [134,135,136]. It is worthy of note that SCC regresses in patients treated with IFNγ [137]. Despite this clinical evidence, however, IFNγ is known to synergize with IL-1 and TNF at promoting EMT and cellular invasion [138,139]. In fact, exposure of either normal or tumor epithelial cells to IL-1, TNFα, or IFNγ upregulates the expression of the EGF receptor (EGFR) (Figure 2) [140,141,142,143,144,145,146]. Specifically concerning cervical epithelial cells, when EGF is released by tissue-infiltrating inflammatory cells [147], it binds to EGFR driving glycogen synthase kinase 3β inactivation, and the consequent upregulation and nuclear accumulation of EMT-promoting transcription factors (Figure 2) [148,149].

As for inflammatory cytokines, the expression of EGFR increases from L-SIL to H-SIL and from the latter to SCC, as compared with the healthy cervical epithelium [148,150,151]. This phenomenon coincides with the fact that, while in healthy cervical epithelium EGFR is expressed almost exclusively in the basal layer, in SIL and especially in SCC the EGFR is present in all layers, these being infiltrated with migrating and proliferating basal cells [62,71,72]. In this regard, it has to be highlighted that, besides directly upregulating EGFR, the inflammatory IL-1, IL-6, TNFα, or IFNγ promotes epithelial cell migration [152,153,154,155]. Taken together, these results describe one of the many cases in which inflammation favors cancer onset or progression, rather than counteracting them [117].

Still about this, it is noteworthy that in addition to EGFR, the TNFα, IL-1, and/or IFNγ upregulate also the expression of TGF-β1 and its type I and II receptors (Figure 2) [139,156,157,158,159]. In this regard, one should consider that TGF-β1 is arguably the most powerful inducer of EMT in both normal and transformed epithelial cells [3,13,148,160,161,162,163,164,165,166]. In particular, following its release by inflammatory leukocytes, TGF-β1 binds to, and phosphorylates, the TGF-β receptor II expressed on the surface of either normal or tumor epithelial cells [166,167]. Upon its activation, the TGF-β receptor II phosphorylates the TGF-β receptor I, this leading to the aggregation of the Smad 2, 3, and 4 cytoplasmic proteins into a trimeric complex which enters the nucleus, thereby promoting Snail, Zeb1, Zeb2, or Twist gene expression (Figure 2) [166,167].

Similar to what happens for inflammatory cytokines and EGFR, an increase in TGF-β1 expression in the uterine cervix has been shown to accompany L-SIL progression to H-SIL [168,169]. Some authors have reported that the upregulation of TGF-β1 is transient since its levels in the lesions are reduced when H-SIL evolves into SCC [168,169]. In contrast, other studies have found that TGF-β1 protein levels also augment during SIL progression to SCC and that this parallels the upregulation of HPV-E7 [170] which, as specified already, can promote TGF-β1 expression [105].

It is worthy of the greatest interest that, HPV-E5, E6, and E7, as well as IL-1, IL-6, TNFα, IFNγ, EGF, and TGF-β1, are capable of triggering the MAPK/ERK and/or the PI3K/AKT/mTOR pathways, leading to the activation of EMT-promoting transcription factors (Figure 2) [7,9,11,14,68,69,123,146,161,171,172,173,174,175,176,177,178,179,180,181,182,183,184].

Therefore, the aberrant activation of the PI3K/AKT/mTOR and/or MAPK/ERK pathways observed in uterine SIL or SCC [185,186] is most likely due to the concurrent activities of inflammatory mediators, growth factors, and HR-HPV oncoproteins.

The strong activation of AKT and MAPK, and the cross-talks between these signaling pathways, rapidly induce the EMT pro-invasive phenotype in cervical epithelial cells, thereby promoting SIL and its progression to invasive SCC [9,17,79,80,81,82,83,84,85,87,185,186,187]. In addition, activated AKT and MAPK effectively sustain the survival and proliferation of the epithelial–mesenchymal hybrids (Figure 2) [188,189]. These phenomena are magnified when HPV-E6 nullifies p53 ability to arrest the growth or promote the death of cells whose genomic integrity has been compromised by carcinogens, HR-HPVs included: in such an eventuality, epithelial–mesenchymal hybrids with damaged DNA survive and proliferate uncontrollably [107,109,190,191].

## 4. Cyclic Hypoxia Exacerbates EMT and Favors the Appearance of Stem-Like Cells in Cervical Squamous Lesions

**Figure 3:**
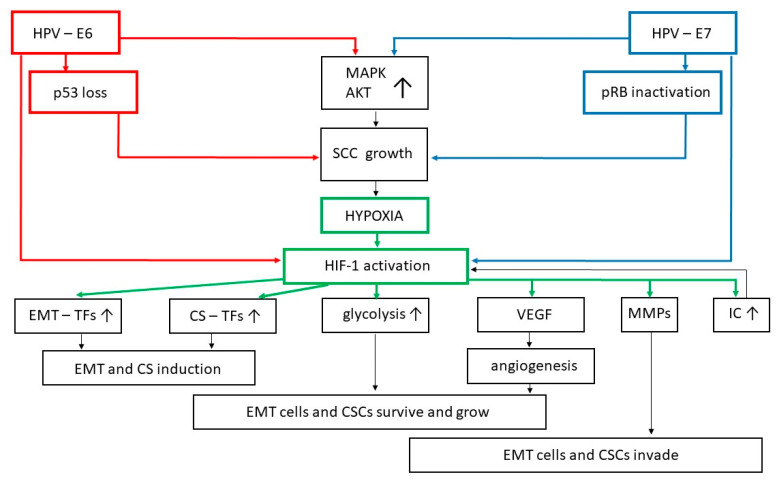
The HPV-E6 and E7 proteins strengthen HIF-1 capability of promoting EMT and cellular stemness. Arrows symbolize directions of connections. Abbreviations: AKT: protein kinase B; CS: cellular stemness; CSC: cancer stem cell; EMT: epithelial-to-mesenchymal transition; HPV: human papillomavirus; IC: inflammatory cytokines; MAPK: mitogen-activated protein kinase; MMP: matrix metalloproteinase; pRb: retinoblastoma protein; SCC: squamous cell carcinoma; TF: transcription factor; VEGF: vascular endothelial growth factor.

During tumor progression, the proliferating cancer cells at the beginning infiltrate the tissue area where they developed, and then move towards its periphery, hence increasing the size of the neoplastic mass: at this point, local vessels cannot satisfy the strong demand that the growing tumor has for oxygen and nutrients [192,193]. As a consequence, an acidified and hypoxic microenvironment is produced leading to the activation of the hypoxia-inducible factor (HIF)-1 (Figure 3) [192,193].

HIF-1 is a transcription factor consisting of a constitutively expressed subunit HIF-1β and an oxygen-regulated subunit HIF-1α [193]. Under normal oxygen tension (normoxia), post-translational modifications of the HIF-1α subunit trigger its degradation via the ubiquitin–proteasome pathway [193]. In hypoxia, HIF-1α is stabilized and interacts with transcriptional coactivators to promote the expression of target genes such as that coding for vascular endothelial growth factor (VEGF)-A: the latter, in turn, stimulates angiogenesis, that is the formation of new blood vessels directed at nourishing the growing tumor (Figure 3) [182,190,192,193].

In the proliferating lesions of the uterine cervix, the expression of HIF is highest in areas that are distant from the pre-existing vessels and close to necrotic zones [194]. Of interest, hypoxic tumor areas are often infiltrated by inflammatory macrophages secreting TNFα, IL-1, and IL-6 which, as cited earlier, are effective inducers of EMT [182].

Between HIF-1 and inflammatory mediators, reciprocal interactions occur, some of which are started or mediated by AKT signaling [195,196]. In particular, HIF-1 directly activates the expression of IL-1, IL-6, or TNF (Figure 3) [196,197,198], as well as that of chemotactic factors recruiting inflammatory cytokine-releasing macrophages [199]. On the other hand, TNFα or IL-6 upregulates HIF-1α gene expression [200,201], while IL-1β increases HIF-1α protein levels by acting post-transcriptionally (Figure 3) [195]. In the HPV-infected cervix, HIF-1α is also stabilized by the E6 and E7 proteins, which block the degradation of HIF-1α protein by the cellular proteasome (Figure 3) [101,102,103,104,202,203,204]. This effect, which again is mediated by E6 or E7 capability of activating the ERK1/2 and PI3K/AKT signaling pathways, leads to an increase in VEGF-mediated tumor angiogenesis (Figure 3), this being particularly evident during H-SIL evolution into invasive SCC [205,206,207]. Lately, the newly formed vessels will provide the tumor with additional metastatic routes [190,192,193].

However, tumor-associated new vessels are highly dysfunctional and, as such, they are not capable of satisfying in full or constantly the great oxygen request by the growing neoplasm [190,192]. As a consequence, phases of hypoxia and normoxia are interspersed in the tumor tissues [190]. During the hypoxic phase, HIF-1 drives EMT in carcinoma cells by activating Snail, Twist, or Zeb expression (Figure 3), either directly or by recruiting the NF-kB transcription factor and/or the TGFβ/Smad signaling pathways [182,190]. When the oxygen level in the tumor tissue returns to normal, HIF is inactivated, and EMT gradually and/or partially converts into MET via ELF3/5, GRHL2, and OVOL1/2 transcriptional activity [190]. This condition, which is termed “cyclic hypoxia”, frequently occurs in SCC tissues [208]: there, the continuous interchange of the epithelial and mesenchymal phenotypes favors the appearance in the lesions, and the persistence therein, of poorly differentiated cells, which resemble stem cells [209,210].

Here it has to be highlighted that in post-natal life stem cells normally reside in specifically dedicated body areas, the basal layer of the multilayered epithelia included: there, stem cells continuously differentiate into mature cells, and self-renew in order to keep their number constant [107,191]. In particular, during physiologic tissue growth or renewal, stem cells replicate each giving rise to two cells of which one remains stem and one differentiates: such a peculiar replication is defined as “asymmetric division”, and it also occurs in the repair of lesions of limited extension [107,191]. In contrast, when the tissue injury is extensive, resident stem cells initially proliferate, each of them eventually giving rise to two stem cells (symmetric division), and so on until some of the numerous stem cells that have been produced differentiate into two mature cells [191].

The balance between pro-differentiation and antidifferentiation stimuli that a stem cell receives from the microenvironment is modulated by a variety of transcription factors. Among them, HIF-1 is very effective as it promptly stimulates the Oct4 and Sox2 transcription factors at inducing the stem cell phenotype (Figure 3) [107]; at the same time, HIF-1 favors stem cell survival by promoting lactate production via glycolysis (Figure 3), a metabolic program which is exacerbated in both stem and cancer cells, rendering them less dependent on oxygen supply than non-transformed, well-differentiated cells [54,211].

In addition to HIF-1 activation promoted by local hypoxia, the expression of stemness markers in cervical squamous lesions could result from the degradation of p53 driven by the E6 protein of HR-HPVs [51]. This is because functional p53 represses the expression of stem cell factors including Oct4, KLF4, LIN28A, Sox2, and c-myc by acting on them directly or through the activity of miRs, such as miR-34a and miR-145 (Figure 1) [34,191,212,213]. Furthermore, inhibitory interactions exist between p53 and other stemness-related genes such as STAT3 and Piwi-l [214,215].

Additionally, the inflammatory mediators or growth factors with a role in cervical carcinogenesis may contribute to the induction of stemness by activating PI3K/AKT/mTOR or MAPK-ERK and, consequently, Zeb, Snail, and Twist transcriptional activity, which includes the expression of stemness markers (Figure 1, Figure 2 and Figure 3) [34,107,109,191]. In addition, activated AKT or MAPK upregulates the human/murine double minute (H/MDM) 2 protein (Figure 1) which, by hindering p53 transactivation activity, induces stemness and/or causes stem cells to exit quiescence and progress through the cell cycle, thereby triggering stem cell expansion [107]. However, H/MDM2 is also capable of driving Slug degradation [109]. Therefore, because of this dual effect, H/MDM2 is likely to have an important role in the modulation of epithelial cell plasticity.

In view of the impact that HIF-1, p53, Zeb, Snail, and Twist have on EMT, it is easy to understand why cells displaying EMT features together with stem cell markers including nestin, aldehyde dehydrogenase 1, the cholesterol-binding CD133, and the glycosaminoglycan receptor CD44 are present in uterine cervical SCC lesions [88,89,90,92,96,97].

When a malignant transformation is achieved, carcinoma cells may lose any index of differentiation and acquire decidedly stem characteristics (Figure 1, Figure 2 and Figure 3) [216,217]. These cells, termed cancer stem cells (CSCs), are very invasive and vital, being capable of self-renewal like normal stem cells [94,107,191,216,218,219]. Of interest, CSCs are found in uterine SCC from its early stage of development (i.e., H-SIL or SCC in situ), so much so that they have been proposed as markers for the preventive diagnosis of this tumor [90,91,92,94,95,220,221].

The CSCs, whose number in the SCC lesions directly parallels the tumor grade [91,92,94,95,222] are believed to arise from the dedifferentiation of mutated somatic cells and/or the transformation of stem cells [107]: both of these events are largely attributable to the loss of p53 which is undoubtedly one of the key steps in HR-HPV-promoted cervical carcinogenesis. In fact, given that functional p53 guarantees stem cells differentiation and prevent the reconversion of differentiated cells into stem cells, p53 loss causes terminally differentiated somatic cells to revert to stem-like and proliferate; likewise, the functional impairment of p53 triggers adult stem cells to acquire pluripotency and to undergo symmetric divisions [107,191].

In this context, one should consider that markers of EMT and stemness characterize not only uterine CSCs but also the stem cells residing in the basal layer of the cervical epithelium [91,93,94,95,218]. This has suggested that uterine SCC could be initiated by the transformation or the abnormal activation of basal stem cells [223,224]. Such a hypothesis is supported by the finding that the “transformation zone”, that is the area of the cervix in which SCC develops more frequently is particularly rich in stem cells [58,60,61,62].

## 5. EMT and Cellular Stemness Not Only Facilitate SCC Cells Invasion and Spreading, but also Increase SCC Cells Resistance to Anticancer Chemo- or Radiotherapy

Invasive uterine SCC is started when carcinoma cells degrade the basement membrane of the cervical epithelium and penetrate into the underlying stroma [78]. Tissue-infiltrating abilities are especially evident in carcinoma cells that have undergone EMT [187,209,210,225]. In this context, one should consider that Twist, Snail, and other EMT-promoting transcription factors activate the expression of basement membrane-degrading proteolytic enzymes [226,227,228,229]. Among them, the matrix metalloproteinases (MMPs) are pivotal to SCC cell invasion (Figure 1, Figure 2 and Figure 3) [230]. It is worth noting that two members of the MMP family, MMP-2 and MMP-9, are considered as SCC prognostic markers since their expression level positively correlates with the progression of L-SIL to H-SIL, pre-invasive SCC, and, finally, invasive SCC [230].

It has to be highlighted that the EMT transcription factors can promote MMP expression also in an indirect fashion, that is by downregulating E-cadherin and thereby disassembling the intercellular junctions constituted by E-cadherin/cytoplasmic β-catenin complexes: this event, in turn, causes the translocation of cytoplasmic β-catenin to the nucleus where it cooperates with NF-kB at activating MMPs expression [20,231].

The β-catenin is the fundamental component of the wingless-type mouse mammary tumor virus integration site (Wnt) pathway, a signaling axis important to tissue homeostasis because of its impact on cell survival, proliferation, differentiation, and locomotion [232]. It is noteworthy that a deregulated activity of Wnt/β-catenin participates in the onset and progression of uterine cervical SCC where it associates with EMT [233,234]. This is easily predictable given that β-catenin transcriptional activity is stimulated by signaling pathways that are strongly activated by EMT-promoting factors with a role in cervical carcinogenesis, including IL-1, IL-6, TNFα, EGF, and TGF-β1 (Figure 2) [235,236,237,238,239,240,241,242,243,244,245,246,247,248]. Specifically regarding TGF-β1, the most powerful promoter of EMT in uterine epithelial cells, an increase in its levels such that occurring in cervical squamous lesions causes in epithelial cells an abnormal activation of the Smad proteins and PI3K/AKT/mTOR or MAPK/ERK signaling, which synergize at promoting β-catenin nuclear translocation, MMP expression and cellular invasion (Figure 2) [13,160,161,162,163,164,165,171,179,183,249,250,251,252,253]. For its part, EGF cooperates with TGF-β1 at promoting MMP expression and cellular invasiveness (Figure 2) [254]. These molecular events, which confer a pro-oncogenic phenotype to the epithelial–mesenchymal hybrids [171,179,183,251,253], are particularly stressed in HR-HPV-infected cells, where the E6 and E7 proteins downregulate Wnt/β-catenin inhibitors such as the miR-34a and the Na+/H+ exchanger tegulatory factor 1 protein [255,256].

As for the EMT canonical transcription factors and the Wnt/β-catenin axis, HIF is also capable of inducing the expression of MMPs (Figure 3), as well as further extracellular matrix-degrading enzymes [182,257]. Indeed, hypoxia is deeply involved in tumor evolution from the pre-invasive to the invasive phase [258]. In particular, when compared with well-oxygenated SCCs, the SCCs undergoing cyclic hypoxia show a high probability of metastasizing, this being due to their richness in epithelial–mesenchymal hybrids [209,210].

Upon basement membrane degradation, cancer cells reach the stroma driven by growth factors or nutrients [190]. When cells from the primary tumor invade the surrounding stroma, cellular hybrids in which the mesenchymal characters are more expressed than the epithelial ones are present in carcinoma invasive front, where they act as “leader” cells [190]. In contrast, hybrids in which epithelial markers are more abundant than the mesenchymal ones aggregate to each other and follow the leading cells in the invasion path [190]. In accordance with their EMT phenotype, invading leader cells synthesize a new matrix rich in fibronectin and other non-collagenous glycoproteins [259]: such a “soft” matrix does not hinder cellular locomotion but, on the contrary, favors it by providing the cells with mechanical support [21,260].

Cancer cells move through the tissues via the use of long protrusions of the cell membrane and cytosol which result from the polymerization of actin and the maturation of an actin-myosin contractile apparatus [261]. Such phenotypic changes are triggered by Twist 1 or other EMT transcription factors upon their activation by EGF and/or TGF [261]. The rearrangement of the cytoskeleton is followed by the recruitment of MMPs and CD44, both expressed by the uterine cervical SCC cells [88,230], to the forming protrusions: there, CD44 and the MMPs may aggregate, giving rise to macromolecular complexes which on one side mediate cancer cell adhesion to the extracellular matrix, and on the other induce matrix degradation [261].

Migrating leader cells need a lot of energy, which they obtain from glycolysis [190]: as discussed before, glycolysis is strongly stimulated in cellular hybrids upon HIF-1 activation (Figure 3) [54,211]. Once more, the phenotypic plasticity of the epithelial–mesenchymal hybrids favors their locomotion. In fact, a leader cell that has consumed all the available energy is replaced by a follower cell, full of energy, which then takes on more decidedly mesenchymal characteristics [190].

When they reach lymphatic or blood vessels, tumor cells adhere to the vascular wall and, again because of the activity of MMPs and other proteolytic enzymes, degrade it and intravasate [258]. Indeed, SCC cells can be isolated from the blood of patients with advanced cervical cancer [262,263,264]. It is noteworthy that the number of circulating SCC cells is predictive of tumor metastases and/or it inversely correlates with patients’ disease-free survival [263,264]. However, only a small percentage of the tumor cells that circulate in the blood or lymph can resist the apoptosis resulting from the lack of the solid support provided by the extracellular matrix, which epithelial cells, including carcinoma cells, need to survive [21,190]. It is relevant that most of the surviving cells are epithelial–mesenchymal hybrids or CSCs: this is because both phenotypes imply the strong activation of pro-survival signaling pathways including PI3K/AKT, NF-kB, and Wnt/β-catenin, and the inactivation of the pro-apoptosis proteins, such as p53 [21,190,258]. In this regard, it is interesting to note that many of the circulating trans-differentiated tumor cells overexpress EGFR, the activation of which is known to strongly support epithelial cell survival [265,266]. Moreover, because of their ability to aggregate with platelets, circulating cellular hybrids and CSCs are protected from either the stress generated by blood flow turbulence or from the attack by immunocompetent cells [190,267].

When circulating tumor cells reach a vessel whose caliber is smaller than the diameter of the metastatic embolus, they stop, adhere to endothelial cells, degrade the vessel wall, and pass into the extravascular territory which may eventually become the site of metastasis [190,268]. The new environment can be hostile to metastasized cells, as it often differs from the one in which the tumor has originated; nonetheless, the phenotypic plasticity of metastasized cells favors their survival [190]. In fact, once the cancerous epithelial–mesenchymal hybrids have arrived in the secondary site, some of them undergo a MET, returning to assume the original epithelial phenotype [190,258]. While EMT is associated with SCC initiation, invasion, and spreading, MET favors the anchorage-dependent growth of carcinoma cells [269].

However, the EMT to MET switch occurs slowly and often incompletely, causing tumor epithelial–mesenchymal hybrids and CSCs to persist in cervical SCC metastases: this is particularly evident in hypoxic areas where activated HIF-1 stimulates glycolysis at levels that guarantee the tumor cell hybrids and CSCs a certain independence from the vessels of the metastatic site [88,209,210,222,270,271].

From a medical point of view, it is of concern that CSCs are inherently protected from apoptosis triggered by the DNA-damaging chemotherapeutic or radiations [88,222,272]. Once again, this results from the constitutive activation of AKT or Wnt signaling, or from E6-promoted p53 degradation leading to the upregulation of p53-repressed survival factors, such as Bcl-2 [21,51,94,107,190,216,218,219,258].

Indeed, clinical data indicate a poor prognosis for patients affected by uterine cervical SCC rich in epithelial–mesenchymal hybrids and CSCs [92,271,272,273]. This is particularly evident in scarcely oxygenated uterine SCCs where HIF-1 is activated to promote EMT and stemness [209,210,270]. Most disappointingly, radiotherapy worsens the situation. In fact, the proportion of CSCs over total cancer cell number in cervical SCC tissues increases after radiation therapy [97], likely because of the radiations-induced vessel damage, and the resultant hypoxia [274].

## 6. Concluding Remarks and Future Directions

SCC of the uterine cervix is a leading cause of death among women worldwide, although its onset can be prevented in the first instance by anti-HPV vaccination [275] and, in the second instance, through screening programs for the detection and surveillance of squamous precancerous lesions [58,276]. If H-SIL is present, its surgical removal will be advisable which, however, does not cancel the possibility of relapse [276].

Concerning the treatment of advanced cervical SCC, the failure of conventional antineoplastic therapy invokes the design and evaluation of novel therapeutic approaches. Given the role that EMT plays in the onset, progression, or metastatization of SCC, and in its resistance to anti-tumor chemotherapy or radiations, particular attention should be paid to drugs countering the EMT process and possibly eradicating CSCs within cervical SCC.

In this regard, it has to be highlighted that switching off the PI3K/AKT/mTOR, MAPK/ERK, or Wnt/β-catenin signaling pathways can revert EMT to MET and halt tumor cell invasion [107,232,234,277,278,279,280]. In addition, antagonists of hypoxia-responsive genes have been shown to inhibit SCC cell invasion [281]. Moreover, H/MDM2 inhibitors can promote the differentiation of CSCs and, at the same time, augment their sensitivity to conventional cytotoxic drugs [107]. Similarly, a restored expression of p53-transcribed miRs, such as miR-34a and miR-203, diminishes cancer cell resistance to chemotherapeutic agents [34,51].

Definitely, a deeper understanding of the interplay among the HR-HPV proteins, oncosuppressor genes, cellular regulators of gene expression, inflammatory mediators, and growth factors involved in cervical carcinogenesis could provide clues to developing new strategies hindering the onset, growth, metastatization, or recurrence of uterine SCC.

## Figures and Tables

**Table 1:** The HR-HPV-E5 protein: activities and biological effects with a role in cervical carcinogenesis.

E5 Activity	Effect on HPV-Infected EC
Inhibition of EGFR degradation, enhancement of ET-1 growth effect, p21 and p27 downregulation, cx43 counteraction	Proliferation
Downregulation of the expression of epithelial FGFR2b	Lack of differentiation
Reduction in CD1d and MHC levels on the plasma membrane	Impaired clearance by immune cells
Fas downregulation and Bax degradation	Survival
Induction of the expression of mesenchymal FGFR2c, activation of AKT and MAPK	EMT and tumorigenic behavior

## Abbreviations

AKT	protein kinase B
CSC	cancer stem cell
E-cadherin	epithelial-cadherin
EGF	epidermal growth factor
EGFR	epidermal growth factor receptor
EMT	epithelial-to-mesenchymal transition
ERK	extracellular-regulated kinase
FGFR	fibroblast growth factor receptor
HIF	hypoxia-inducible factor
HLA	human leukocyte antigen
H/MDM2	human/murine double minute 2
HR-HPV	high-risk human papillomavirus
H-SIL	high-grade squamous intraepithelial lesion
IFN	interferon
IL	interleukin
L-SIL	low-grade squamous intraepithelial lesion
MAPK	mitogen-activated protein kinase
MET	mesenchymal-to-epithelial transition
MHC	major histocompatibility complex
miR	microRNA
MMP	matrix metalloproteinase
mTOR	mammalian target of rapamycin
N-cadherin	neuronal-cadherin
NF-kB	nuclear factor-kappa B
PI3K	phosphoinositide-3-kinase
pRb	retinoblastoma protein
SCC	squamous cell carcinoma
SIL	squamous intraepithelial lesion
TGF	transforming growth factor
TNF	tumor necrosis factor
VEGF	vascular endothelial growth factor
Wnt	wingless-type mouse mammary tumor virus integration site
Zeb	zinc finger E-box-binding homeobox

## References

[1] Haensel D., Dai X. (2018). Epithelial-to-mesenchymal transition in cutaneous wound healing: Where we are and where we are heading. Dev. Dyn..

[2] Wang P.H., Huang B.S., Horng H.C., Yeh C.C., Chen Y.J. (2018). Wound healing. J. Chin. Med. Assoc..

[3] Lamouille S., Xu J., Derynck R. (2014). Molecular mechanisms of epithelial-mesenchymal transition. Nat. Rev. Mol. Cell Biol..

[4] Sha Y., Haensel D., Gutierrez G., Du H., Dai X., Nie Q. (2019). Intermediate cell states in epithelial-to-mesenchymal transition. Phys. Biol..

[5] Zhang Y., Weinberg R.A. (2018). Epithelial-to-mesenchymal transition in cancer: Complexity and opportunities. Front. Med..

[6] Liarte S., Bernabé-García Á., Nicolás F.J. (2020). Human Skin Keratinocytes on Sustained TGF-β Stimulation Reveal Partial EMT Features and Weaken Growth Arrest Responses. Cells.

[7] Elsum I.A., Martin C., Humbert P.O. (2013). Scribble regulates an EMT polarity pathway through modulation of MAPK-ERK signaling to mediate junction formation. J. Cell Sci..

[8] Tanahashi T., Osada S., Yamada A., Kato J., Yawata K., Mori R., Imai H., Sasaki Y., Saito S., Tanaka Y. (2013). Extracellular signal-regulated kinase and Akt activation play a critical role in the process of hepatocyte growth factor-induced epithelial-mesenchymal transition. Int. J. Oncol..

[9] Carpenter R.L., Paw I., Dewhirst M.W., Lo H.W. (2015). Akt phosphorylates and activates HSF-1 independent of heat shock, leading to Slug overexpression and epithelial-mesenchymal transition (EMT) of HER2-overexpressing breast cancer cells. Oncogene.

[10] Tang H., Massi D., Hemmings B.A., Mandalà M., Hu Z., Wicki A., Xue G. (2016). AKT-ions with a TWIST between EMT and MET. Oncotarget.

[11] Liu J.Y., Jiang L., He T., Liu J.J., Fan J.Y., Xu X.H., Tang B., Shi Y., Zhao Y.L., Qian F. (2019). NETO2 promotes invasion and metastasis of gastric cancer cells via activation of PI3K/Akt/NF-κB/Snail axis and predicts outcome of the patients. Cell Death Dis..

[12] Hagemann T., Bozanovic T., Hooper S., Ljubic A., Slettenaar V.I., Wilson J.L., Singh N., Gayther S.A., Shepherd J.H., van Trappen P.O. (2007). Molecular profiling of cervical cancer progression. Br. J. Cancer.

[13] Hatta M., Miyake Y., Uchida K., Yamazaki J. (2018). Keratin 13 gene is epigenetically suppressed during transforming growth factor-β1-induced epithelial-mesenchymal transition in a human keratinocyte cell line. Biochem. Biophys. Res. Commun..

[14] Markopoulos G.S., Roupakia E., Marcu K.B., Kolettas E. (2019). Epigenetic Regulation of Inflammatory Cytokine-Induced Epithelial-To-Mesenchymal Cell Transition and Cancer Stem Cell Generation. Cells.

[15] Lin Y.T., Wu K.J. (2020). Epigenetic regulation of epithelial-mesenchymal transition: Focusing on hypoxia and TGF-β signaling. J. Biomed. Sci..

[16] Georgakopoulos-Soares I., Chartoumpekis D.V., Kyriazopoulou V., Zaravinos A. (2020). EMT Factors and Metabolic Pathways in Cancer. Front. Oncol..

[17] Zhang W.N., Li W., Wang X.L., Hu Z., Zhu D., Ding W.C., Liu D., Li K.Z., Ma D., Wang H. (2016). CLDN1 expression in cervical cancer cells is related to tumor invasion and metastasis. Oncotarget.

[18] Lv J., Sun B., Mai Z., Jiang M., Du J. (2017). CLDN-1 promoted the epithelial to migration and mesenchymal transition (EMT) in human bronchial epithelial cells via Notch pathway. Mol. Cell. Biochem..

[19] Nishimoto Y., Murakami A., Sato S., Kajimura T., Nakashima K., Yakabe K., Sueoka K., Sugino N. (2018). Decreased carbonyl reductase 1 expression promotes tumor growth via epithelial mesenchymal transition in uterine cervical squamous cell carcinomas. Reprod. Med. Biol..

[20] Kang S.U., Choi J.W., Chang J.W., Kim K.I., Kim Y.S., Park J.K., Kim Y.E., Lee Y.S., Yang S.S., Kim C.H. (2017). N2 non-thermal atmospheric pressure plasma promotes wound healing in vitro and in vivo: Potential modulation of adhesion molecules and matrix metalloproteinase-9. Exp. Dermatol..

[21] Banyard J., Bielenberg D.R. (2015). The role of EMT and MET in cancer dissemination. Connect. Tissue Res..

[22] Bracken C.P., Gregory P.A., Kolesnikoff N., Bert A.G., Wang J., Shannon M.F., Goodall G.J. (2008). A double-negative feedback loop between ZEB1-SIP1 and the microRNA-200 family regulates epithelial-mesenchymal transition. Cancer Res..

[23] Burk U., Schubert J., Wellner U., Schmalhofer O., Vincan E., Spaderna S., Brabletz T. (2008). A reciprocal repression between ZEB1 and members of the miR-200 family promotes EMT and invasion in cancer cells. EMBO Rep..

[24] Siemens H., Jackstadt R., Hünten S., Kaller M., Menssen A., Götz U., Hermeking H. (2011). miR-34 and SNAIL form a double-negative feedback loop to regulate epithelial-mesenchymal transitions. Cell Cycle.

[25] Chakrabarti R., Hwang J., Andres Blanco M., Wei Y., Lukačišin M., Romano R.A., Smalley K., Liu S., Yang Q., Ibrahim T. (2012). Elf5 inhibits the epithelial-mesenchymal transition in mammary gland development and breast cancer metastasis by transcriptionally repressing Snail2. Nat. Cell Biol..

[26] Cieply B., Farris J., Denvir J., Ford H.L., Frisch S.M. (2013). Epithelial-mesenchymal transition and tumor suppression are controlled by a reciprocal feedback loop between ZEB1 and Grainyhead-like-2. Cancer Res..

[27] Roca H., Hernandez J., Weidner S., McEachin R.C., Fuller D., Sud S., Schumann T., Wilkinson J.E., Zaslavsky A., Li H. (2013). Transcription factors OVOL1 and OVOL2 induce the mesenchymal to epithelial transition in human cancer. PLoS ONE.

[28] Jolly M.K., Jia D., Boareto M., Mani S.A., Pienta K.J., Ben-Jacob E., Levine H. (2015). Coupling the modules of EMT and stemness: A tunable ‘stemness window’ model. Oncotarget.

[29] Jolly M.K., Tripathi S.C., Jia D., Mooney S.M., Celiktas M., Hanash S.M., Mani S.A., Pienta K.J., Ben-Jacob E., Levine H. (2016). Stability of the hybrid epithelial/mesenchymal phenotype. Oncotarget.

[30] Chung V.Y., Tan T.Z., Tan M., Wong M.K., Kuay K.T., Yang Z., Ye J., Muller J., Koh C.M., Guccione E. (2016). GRHL2-miR-200-ZEB1 maintains the epithelial status of ovarian cancer through transcriptional regulation and histone modification. Sci. Rep..

[31] Mooney S.M., Talebian V., Jolly M.K., Jia D., Gromala M., Levine H., McConkey B.J. (2017). The GRHL2/ZEB Feedback Loop-A Key Axis in the Regulation of EMT in Breast Cancer. J. Cell Biochem..

[32] Liu D., Skomorovska Y., Song J., Bowler E., Harris R., Ravasz M., Bai S., Ayati M., Tamai K., Koyuturk M. (2019). ELF3 is an antagonist of oncogenic-signalling-induced expression of EMT-TF ZEB1. Cancer Biol. Ther..

[33] Morel A.P., Hinkal G.W., Thomas C., Fauvet F., Courtois-Cox S., Wierinckx A., Devouassoux-Shisheboran M., Treilleux I., Tissier A., Gras B. (2012). EMT inducers catalyze malignant transformation of mammary epithelial cells and drive tumorigenesis towards claudin-low tumors in transgenic mice. PLoS Genet..

[34] Parfenyev S., Singh A., Fedorova O., Daks A., Kulshreshtha R., Barlev N.A. (2021). Interplay between p53 and non-coding RNAs in the regulation of EMT in breast cancer. Cell Death Dis..

[35] Ma L., Young J., Prabhala H., Pan E., Mestdagh P., Muth D., Teruya-Feldstein J., Reinhardt F., Onder T.T., Valastyan S. (2010). miR-9, a MYC/MYCN-activated microRNA, regulates E-cadherin and cancer metastasis. Nat. Cell Biol..

[36] Liao T.T., Yang M.H. (2017). Revisiting epithelial-mesenchymal transition in cancer metastasis: The connection between epithelial plasticity and stemness. Mol. Oncol..

[37] Yokoyama K., Kamata N., Fujimoto R., Tsutsumi S., Tomonari M., Taki M., Hosokawa H., Nagayama M. (2003). Increased invasion and matrix metalloproteinase-2 expression by Snail-induced mesenchymal transition in squamous cell carcinomas. Int. J. Oncol..

[38] Jordà M., Olmeda D., Vinyals A., Valero E., Cubillo E., Llorens A., Cano A., Fabra A. (2005). Upregulation of MMP-9 in MDCK epithelial cell line in response to expression of the Snail transcription factor. J. Cell Sci..

[39] Zhao X., Sun T., Che N., Sun D., Zhao N., Dong X., Gu Q., Yao Z., Sun B. (2011). Promotion of hepatocellular carcinoma metastasis through matrix metalloproteinase activation by epithelial-mesenchymal transition regulator Twist1. Cell. Mol. Med..

[40] Zhao M., Hu H.G., Huang J., Zou Q., Wang J., Liu M.Q., Zhao Y., Li G.Z., Xue S., Wu Z.S. (2013). Expression and correlation of Twist and gelatinases in breast cancer. Exp. Ther. Med..

[41] García-Cuellar C.M., Santibáñez-Andrade M., Chirino Y.I., Quintana-Belmares R., Morales-Bárcenas R., Quezada-Maldonado E.M., Sánchez-Pérez Y. (2021). Particulate Matter (PM_10_) Promotes Cell Invasion through Epithelial-Mesenchymal Transition (EMT) by TGF-β Activation in A549 Lung Cells. Int. J. Mol. Sci..

[42] Lee A.Y., Fan C.C., Chen Y.A., Cheng C.W., Sung Y.J., Hsu C.P., Kao T.Y. (2015). Curcumin Inhibits Invasiveness and Epithelial-Mesenchymal Transition in Oral Squamous Cell Carcinoma Through Reducing Matrix Metalloproteinase 2, 9 and Modulating p53-E-Cadherin Pathway. Integr. Cancer Ther..

[43] Lu L., Zha Z., Zhang P., Li D., Liu G. (2021). NSE, positively regulated by LINC00657-miR-93-5p axis, promotes small cell lung cancer (SCLC) invasion and epithelial-mesenchymal transition (EMT) process. Int. J. Med. Sci..

[44] Wu W.S., You R.I., Cheng C.C., Lee M.C., Lin T.Y., Hu C.T. (2018). Snail collaborates with EGR-1 and SP-1 to directly activate transcription of MMP 9 and ZEB1. Sci. Rep..

[45] Hseu Y.C., Lin Y.C., Rajendran P., Thigarajan V., Mathew D.C., Lin K.Y., Way T.D., Liao J.W., Yang H.L. (2019). Antrodia salmonea suppresses invasion and metastasis in triple-negative breast cancer cells by reversing EMT through the NF-κB and Wnt/β-catenin signaling pathway. Food Chem. Toxicol..

[46] Yu L., Lu S., Tian J., Ma J., Li J., Wang H., Xu W. (2012). TWIST expression in hypopharyngeal cancer and the mechanism of TWIST-induced promotion of metastasis. Oncol. Rep..

[47] Tian T., Li X., Hua Z., Ma J., Wu X., Liu Z., Chen H., Cui Z. (2017). S100A7 promotes the migration, invasion and metastasis of human cervical cancer cells through epithelial-mesenchymal transition. Oncotarget.

[48] Zheng H., Fu Y., Yang T. (2019). Propofol inhibits proliferation, migration, and invasion of hepatocellular carcinoma cells by downregulating Twist. J. Cell Biochem..

[49] Sonongbua J., Siritungyong S., Thongchot S., Kamolhan T., Utispan K., Thuwajit P., Pongpaibul A., Wongkham S., Thuwajit C. (2020). Periostin induces epithelial to mesenchymal transition via the integrin α5β1/TWIST 2 axis in cholangiocarcinoma. Oncol. Rep..

[50] Yao Y., Bellon M., Shelton S.N., Nicot C. (2012). Tumor suppressors p53, p63TAα, p63TAy, p73α, and p73β use distinct pathways to repress telomerase expression. J. Biol. Chem..

[51] Chen J., Zhao K.N. (2015). HPV-p53-miR-34a axis in HPV-associated cancers. Ann. Transl. Med..

[52] Popper H. (2020). Primary tumor and metastasis-sectioning the different steps of the metastatic cascade. Transl. Lung Cancer Res..

[53] Lin C.C., Cheng T.L., Tsai W.H., Tsai H.J., Hu K.H., Chang H.C., Yeh C.W., Chen Y.C., Liao C.C., Chang W.T. (2012). Loss of the respiratory enzyme citrate synthase directly links the Warburg effect to tumor malignancy. Sci. Rep..

[54] Courtnay R., Ngo D.C., Malik N., Ververis K., Tortorella S.M., Karagiannis T.C. (2015). Cancer metabolism and the Warburg effect: The role of HIF-1 and PI3K. Mol. Biol. Rep..

[55] Tripathi D., Kulkarni S. (2021). Butein induces intrinsic pathway of apoptosis, vimentin proteolysis, and inhibition of cancer stem cell population in a human papillary thyroid cancer cell line. Toxicol. In Vitro.

[56] Huang R., Zong X. (2017). Aberrant cancer metabolism in epithelial-mesenchymal transition and cancer metastasis: Mechanisms in cancer progression. Crit. Rev. Oncol. Hematol..

[57] Li H., Zhang W., Yan M., Qiu J., Chen J., Sun X., Chen X., Song L., Zhang Y. (2019). Nucleolar and spindle associated protein 1 promotes metastasis of cervical carcinoma cells by activating Wnt/β-catenin signaling. J. Exp. Clin. Cancer Res..

[58] Stumbar S.E., Stevens M., Feld Z. (2019). Cervical Cancer and Its Precursors: A Preventative Approach to Screening, Diagnosis, and Management. Prim. Care.

[59] Fidan U., Keskin U., Ulubay M., Öztürk M., Bodur S. (2017). Value of vaginal cervical position in estimating uterine anatomy. Clin. Anat..

[60] Reich O., Fritsch H. (2014). The developmental origin of cervical and vaginal epithelium and their clinical consequences: A systematic review. J. Low. Genit. Tract Dis..

[61] Yang E.J., Quick M.C., Hanamornroongruang S., Lai K., Doyle L.A., McKeon F.D., Xian W., Crum C.P., Herfs M. (2015). Microanatomy of the cervical and anorectal squamocolumnar junctions: A proposed model for anatomical differences in HPV-related cancer risk. Mod. Pathol..

[62] Doorbar J., Griffin H. (2019). Refining our understanding of cervical neoplasia and its cellular origins. Papillomavirus Res..

[63] Bodily J., Laimins L.A. (2011). Persistence of human papillomavirus infection: Keys to malignant progression. Trends Microbiol..

[64] Latsuzbaia A., Wienecke-Baldacchino A., Tapp J., Arbyn M., Karabegović I., Chen Z., Fischer M., Mühlschlegel F., Weyers S., Pesch P. (2020). Characterization and Diversity of 243 Complete Human Papillomavirus Genomes in Cervical Swabs Using Next Generation Sequencing. Viruses.

[65] Vande Pol S.B., Klingelhutz A.J. (2013). Papillomavirus E6 oncoproteins. Virology.

[66] Hoppe-Seyler K., Bossler F., Braun J.A., Herrmann A.L., Hoppe-Seyler F. (2018). The HPV E6/E7 Oncogenes: Key Factors for Viral Carcinogenesis and Therapeutic Targets. Trends Microbiol..

[67] Jiang Z., Albanese J., Kesterson J., Warrick J., Karabakhtsian R., Dadachova E., Phaëton R. (2019). Monoclonal Antibodies Against Human Papillomavirus E6 and E7 Oncoproteins Inhibit Tumor Growth in Experimental Cervical Cancer. Transl. Oncol..

[68] Venuti A., Paolini F., Nasir L., Corteggio A., Roperto S., Campo M.S., Borzacchiello G. (2011). Papillomavirus E5: The smallest oncoprotein with many functions. Mol. Cancer.

[69] Basukala O., Banks L. (2021). The Not-So-Good, the Bad and the Ugly: HPV E5, E6 and E7 Oncoproteins in the Orchestration of Carcinogenesis. Viruses.

[70] Ranieri D., French D., Raffa S., Guttieri L., Torrisi M.R., Belleudi F. (2021). Expression of the E5 Oncoprotein of HPV16 Impacts on the Molecular Profiles of EMT-Related and Differentiation Genes in Ectocervical Low-Grade Lesions. Int. J. Mol. Sci..

[71] Jenkins D. (2007). Histopathology and cytopathology of cervical cancer. Dis. Markers.

[72] Petignat P., Roy M. (2007). Diagnosis and management of cervical cancer. BMJ.

[73] Horn L.C., Klostermann K. (2011). Precancerous lesions of the uterine cervix: Morphology and molecular pathology. Pathologe.

[74] Grabowska A.K., Riemer A.B. (2012). The invisible enemy—How human papillomaviruses avoid recognition and clearance by the host immune system. Open Virol. J..

[75] De Freitas A.C., de Oliveira T.H.A., Barros M.R., Venuti A. (2017). hrHPV E5 oncoprotein: Immune evasion and related immunotherapies. J. Exp. Clin. Cancer Res..

[76] Mittal S., Basu P., Muwonge R., Banerjee D., Ghosh I., Sengupta M.M., Das P., Dey P., Mandal R., Panda C. (2017). Risk of high-grade precancerous lesions and invasive cancers in high-risk HPV-positive women with normal cervix or CIN 1 at baseline-A population-based cohort study. Int. J. Cancer.

[77] McBride A.A., Warburton A. (2017). The role of integration in oncogenic progression of HPV-associated cancers. PLoS Pathog..

[78] Day E., Duffy S., Bryson G., Syed S., Shanbhag S., Burton K., Lindsay R., Siddiqui N., Millan D. (2016). Multifocal FIGO Stage IA1 Squamous Carcinoma of the Cervix: Criteria for Identification, Staging, and its Good Clinical Outcome. Int. J. Gynecol. Pathol..

[79] Li Y., Wang W., Wang W., Yang R., Wang T., Su T., Weng D., Tao T., Li W., Ma D. (2012). Correlation of TWIST2 up-regulation and epithelial-mesenchymal transition during tumorigenesis and progression of cervical carcinoma. Gynecol. Oncol..

[80] Chen Z., Li S., Huang K., Zhang Q., Wang J., Li X., Hu T., Wang S., Yang R., Jia Y. (2013). The nuclear protein expression levels of SNAI1 and ZEB1 are involved in the progression and lymph node metastasis of cervical cancer via the epithelial-mesenchymal transition pathway. Hum. Pathol..

[81] Zhao W., Zhou Y., Xu H., Cheng Y., Kong B. (2013). Snail family proteins in cervical squamous carcinoma: Expression and significance. Clin. Investig. Med..

[82] Wang T., Li Y., Wang W., Tuerhanjiang A., Wu Z., Yang R., Yuan M., Ma D., Wang W., Wang S. (2014). Twist2, the key Twist isoform related to prognosis, promotes invasion of cervical cancer by inducing epithelial-mesenchymal transition and blocking senescence. Hum. Pathol..

[83] Liu Y., Qian W., Zhang J., Dong Y., Shi C., Liu Z., Wu S. (2015). The indicative function of Twist2 and E-cadherin in HPV oncogene-induced epithelial-mesenchymal transition of cervical cancer cells. Oncol. Rep..

[84] Ma Y., Zheng X., Zhou J., Zhang Y., Chen K. (2015). ZEB1 promotes the progression and metastasis of cervical squamous cell carcinoma via the promotion of epithelial-mesenchymal transition. Int. J. Clin. Exp. Pathol..

[85] Ran J., Lin D.L., Wu R.F., Chen Q.H., Huang H.P., Qiu N.X., Quan S. (2015). ZEB1 promotes epithelial-mesenchymal transition in cervical cancer metastasis. Fertil. Steril..

[86] Yang H., Hu H., Gou Y., Hu Y., Li H., Zhao H., Wang B., Li P., Zhang Z. (2018). Combined detection of Twist1, Snail1 and squamous cell carcinoma antigen for the prognostic evaluation of invasion and metastasis in cervical squamous cell carcinoma. Int. J. Clin. Oncol..

[87] Yu J.Q., Zhou Q., Zheng Y.F., Bao Y. (2015). Expression of Vimentin and Ki-67 Proteins in Cervical Squamous Cell Carcinoma and their Relationships with Clinicopathological Features. Asian Pac. J. Cancer Prev..

[88] Feng D., Peng C., Li C., Zhou Y., Li M., Ling B., Wei H., Tian Z. (2009). Identification and characterization of cancer stem-like cells from primary carcinoma of the cervix uteri. Oncol. Rep..

[89] Yao T., Chen Q., Zhang B., Zhou H., Lin Z. (2011). The expression of ALDH1 in cervical carcinoma. Med. Sci. Monit..

[90] Bae H.S., Chung Y.W., Lee J.K., Lee N.W., Yeom B.W., Lee K.W., Song J.Y. (2013). Nestin expression as an indicator of cervical cancer initiation. Eur. J. Gynaecol. Oncol..

[91] Gao Q., Liu W., Cai J., Li M., Gao Y., Lin W., Li Z. (2014). EphB2 promotes cervical cancer progression by inducing epithelial-mesenchymal transition. Hum. Pathol..

[92] Liu W., Gao Q., Chen K., Xue X., Li M., Chen Q., Zhu G., Gao Y. (2014). Hiwi facilitates chemoresistance as a cancer stem cell marker in cervical cancer. Oncol. Rep..

[93] Liu X.F., Yang W.T., Xu R., Liu J.T., Zheng P.S. (2014). Cervical cancer cells with positive Sox2 expression exhibit the properties of cancer stem cells. PLoS ONE.

[94] Lin J., Liu X., Ding D. (2015). Evidence for epithelial-mesenchymal transition in cancer stem-like cells derived from carcinoma cell lines of the cervix uteri. Int. J. Clin. Exp. Pathol..

[95] Javed S., Sharma B.K., Sood S., Sharma S., Bagga R., Bhattacharyya S., Rayat C.S., Dhaliwal L., Srinivasan R. (2018). Significance of CD133 positive cells in four novel HPV-16 positive cervical cancer-derived cell lines and biopsies of invasive cervical cancer. BMC Cancer.

[96] Zhang Y., An J., Liu M., Li N., Wang W., Yao H., Li N., Yang X., Sun Y., Xu N. (2020). Efficient isolation, culture, purification, and stem cell expression profiles of primary tumor cells derived from uterine cervical squamous cell carcinoma. Am. J. Reprod. Immunol..

[97] Zamulaeva I., Selivanova E., Matchuk O., Kiseleva V., Mkrtchyan L., Krikunova L. (2021). Radiation Response of Cervical Cancer Stem Cells Is Associated with Pretreatment Proportion of These Cells and Physical Status of HPV DNA. Int. J. Mol. Sci..

[98] Liu Y., Wang Y., Chen C., Zhang J., Qian W., Dong Y., Liu Z., Zhang X., Wang X., Zhang Z. (2017). LSD1 binds to HPV16 E7 and promotes the epithelial-mesenchymal transition in cervical cancer by demethylating histones at the Vimentin promoter. Oncotarget.

[99] Carrillo D., Muñoz J.P., Huerta H., Leal G., Corvalán A., León O., Calaf G.M., Urzúa U., Boccardo E., Tapia J.C. (2017). Upregulation of PIR gene expression induced by human papillomavirus E6 and E7 in epithelial oral and cervical cells. Open Biol..

[100] Morgan E.L., Scarth J.A., Patterson M.R., Wasson C.W., Hemingway G.C., Barba-Moreno D., Macdonald A. (2021). E6-mediated activation of JNK drives EGFR signalling to promote proliferation and viral oncoprotein expression in cervical cancer. Cell Death Differ..

[101] Tang X., Zhang Q., Nishitani J., Brown J., Shi S., Le A.D. (2007). Overexpression of human papillomavirus type 16 oncoproteins enhances hypoxia-inducible factor 1 alpha protein accumulation and vascular endothelial growth factor expression in human cervical carcinoma cells. Clin. Cancer Res..

[102] Zhang E., Feng X., Liu F., Zhang P., Liang J., Tang X. (2014). Roles of PI3K/Akt and c-Jun signaling pathways in human papillomavirus type 16 oncoprotein-induced HIF-1α, VEGF, and IL-8 expression and in vitro angiogenesis in non-small cell lung cancer cells. PLoS ONE.

[103] Liu F., Lin B., Liu X., Zhang W., Zhang E., Hu L., Ma Y., Li X., Tang X. (2016). ERK Signaling Pathway Is Involved in HPV-16 E6 but not E7 Oncoprotein-Induced HIF-1α Protein Accumulation in NSCLC Cells. Oncol. Res..

[104] Rho S.B., Lee S.H., Byun H.J., Kim B.R., Lee C.H. (2020). IRF-1 Inhibits Angiogenic Activity of HPV16 E6 Oncoprotein in Cervical Cancer. Int. J. Mol. Sci..

[105] Peralta-Zaragoza O., Bermúdez-Morales V., Gutiérrez-Xicotencatl L., Alcocer-González J., Recillas-Targa F., Madrid-Marina V. (2006). E6 and E7 oncoproteins from human papillomavirus type 16 induce activation of human transforming growth factor beta1 promoter throughout Sp1 recognition sequence. Viral Immunol..

[106] Fan Z., Cui H., Xu X., Lin Z., Zhang X., Kang L., Han B., Meng J., Yan Z., Yan X. (2015). MiR-125a suppresses tumor growth, invasion and metastasis in cervical cancer by targeting STAT3. Oncotarget.

[107] Olivos D.J., Mayo L.D. (2016). Emerging Non-Canonical Functions and Regulation by p53: p53 and Stemness. Int. J. Mol. Sci..

[108] Dong P., Xiong Y., Hanley S.J.B., Yue J., Watari H. (2017). Musashi-2, a novel oncoprotein promoting cervical cancer cell growth and invasion, is negatively regulated by p53-induced miR-143 and miR-107 activation. J. Exp. Clin. Cancer Res..

[109] Oikawa T., Otsuka Y., Sabe H. (2018). p53-Dependent and -Independent Epithelial Integrity: Beyond miRNAs and Metabolic Fluctuations. Cancers.

[110] Wan H.Y., Li Q.Q., Zhang Y., Tian W., Li Y.N., Liu M., Li X., Tang H. (2014). MiR-124 represses vasculogenic mimicry and cell motility by targeting amotL1 in cervical cancer cells. Cancer Lett..

[111] Zhang X., Cai D., Meng L., Wang B. (2016). MicroRNA-124 inhibits proliferation, invasion, migration and epithelial-mesenchymal transition of cervical carcinoma cells by targeting astrocyte-elevated gene-1. Oncol. Rep..

[112] Li G.C., Xin L., Wang Y.S., Chen Y. (2019). Long Intervening Noncoding 00467 RNA Contributes to Tumorigenesis by Acting as a Competing Endogenous RNA against miR-107 in Cervical Cancer Cells. Am. J. Pathol..

[113] Li H., Wang J., Xu F., Wang L., Sun G., Wang J., Yang Y. (2019). By downregulating PBX3, miR-526b suppresses the epithelial-mesenchymal transition process in cervical cancer cells. Future Oncol..

[114] Arima Y., Inoue Y., Shibata T., Hayashi H., Nagano O., Saya H., Taya Y. (2008). Rb depletion results in deregulation of E-cadherin and induction of cellular phenotypic changes that are characteristic of the epithelial-to-mesenchymal transition. Cancer Res..

[115] Rezaei M., Mostafaei S., Aghaei A., Hosseini N., Darabi H., Nouri M., Etemadi A., Neill A.O., Nahand J.S., Mirzaei H. (2020). The association between HPV gene expression, inflammatory agents and cellular genes involved in EMT in lung cancer tissue. BMC Cancer.

[116] Knudsen E.S., McClendon A.K., Franco J., Ertel A., Fortina P., Witkiewicz A.K. (2015). RB loss contributes to aggressive tumor phenotypes in MYC-driven triple negative breast cancer. Cell Cycle.

[117] Dominguez C., David J.M., Palena C. (2017). Epithelial-mesenchymal transition and inflammation at the site of the primary tumor. Semin. Cancer Biol..

[118] Hammes L.S., Tekmal R.R., Naud P., Edelweiss M.I., Kirma N., Valente P.T., Syrjänen K.J., Cunha-Filho J.S. (2007). Macrophages, inflammation and risk of cervical intraepithelial neoplasia (CIN) progression—Clinicopathological correlation. Gynecol. Oncol..

[119] Hiraku Y., Tabata T., Ma N., Murata M., Ding X., Kawanishi S. (2007). Nitrative and oxidative DNA damage in cervical intraepithelial neoplasia associated with human papilloma virus infection. Cancer Sci..

[120] Mhatre M., McAndrew T., Carpenter C., Burk R.D., Einstein M.H., Herold B.C. (2012). Cervical intraepithelial neoplasia is associated with genital tract mucosal inflammation. Sex. Transm Dis..

[121] Saldivar J.S., Lopez D., Feldman R.A., Tharappel-Jacob R., de la Rosa A., Terreros D., Baldwin W.S. (2007). COX-2 overexpression as a biomarker of early cervical carcinogenesis: A pilot study. Gynecol. Oncol..

[122] de Castro-Sobrinho J.M., Rabelo-Santos S.H., Fugueiredo-Alves R.R., Derchain S., Sarian L.O., Pitta D.R., Campos E.A., Zeferino L.C. (2016). Bacterial vaginosis and inflammatory response showed association with severity of cervical neoplasia in HPV-positive women. Diagn. Cytopathol..

[123] Zhang L., Wu J., Ling M.T., Zhao L., Zhao K.N. (2015). The role of the PI3K/Akt/mTOR signalling pathway in human cancers induced by infection with human papillomaviruses. Mol. Cancer.

[124] Miao J.W., Liu L.J., Huang J. (2014). Interleukin-6-induced epithelial-mesenchymal transition through signal transducer and activator of transcription 3 in human cervical carcinoma. Int. J. Oncol..

[125] Carrero Y., Mosquera J., Callejas D., Alvarez-Mon M. (2015). In situ increased chemokine expression in human cervical intraepithelial neoplasia. Pathol. Res. Pract..

[126] Zhang W., Tian X., Mumtahana F., Jiao J., Zhang T., Croce K.D., Ma D., Kong B., Cui B. (2015). The existence of Th22, pure Th17 and Th1 cells in CIN and Cervical Cancer along with their frequency variation in different stages of cervical cancer. BMC Cancer.

[127] de Matos L.G., Cândido E.B., Vidigal P.V., Bordoni P.H., Lamaita R.M., Carneiro M.M., da Silva-Filho A.L. (2017). Association between Toll-like receptor and tumor necrosis factor immunological pathways in uterine cervical neoplasms. Tumori.

[128] Takano H., Harigaya K., Ishii G., Sugaya Y., Soeta S., Nunoyama T., Shirasawa H., Shimizu K., Tokita H., Simizu B. (1996). Interleukin-6 (IL-6) production in carcinoma of the cervix. Arch. Gynecol. Obstet..

[129] Woods K.V., Adler-Storthz K., Clayman G.L., Francis G.M., Grimm E.A. (1998). Interleukin-1 regulates interleukin-6 secretion in human oral squamous cell carcinoma in vitro: Possible influence of p53 but not human papillomavirus E6/E7. Cancer Res..

[130] Ho Y., Wu C.Y., Chin Y.T., Li Z.L., Pan Y.S., Huang T.Y., Su P.Y., Lee S.Y., Crawford D.R., Su K.W. (2020). NDAT suppresses pro-inflammatory gene expression to enhance resveratrol-induced anti-proliferation in oral cancer cells. Food Chem. Toxicol..

[131] Gaiotti D., Chung J., Iglesias M., Nees M., Baker P.D., Evans C.H., Woodworth C.D. (2000). Tumor necrosis factor-alpha promotes human papillomavirus (HPV) E6/E7 RNA expression and cyclin-dependent kinase activity in HPV-immortalized keratinocytes by a ras-dependent pathway. Mol. Carcinog..

[132] Arany I., Muldrow M., Tyring S.K. (2001). Correlation between mRNA levels of IL-6 and TNF alpha and progression rate in anal squamous epithelial lesions from HIV-positive men. Anticancer Res..

[133] Iwata T., Fujii T., Morii K., Saito M., Sugiyama J., Nishio H., Morisada T., Tanaka K., Yaguchi T., Kawakami Y. (2015). Cytokine profile in cervical mucosa of Japanese patients with cervical intraepithelial neoplasia. Int. J. Clin. Oncol..

[134] Scott M.E., Ma Y., Kuzmich L., Moscicki A.B. (2009). Diminished IFN-gamma and IL-10 and elevated Foxp3 mRNA expression in the cervix are associated with CIN 2 or 3. Int. J. Cancer.

[135] Chen Z., Ding J., Pang N., Du R., Meng W., Zhu Y., Zhang Y., Ma C., Ding Y. (2013). The Th17/Treg balance and the expression of related cytokines in Uygur cervical cancer patients. Diagn. Pathol..

[136] Ma D., Jiang C., Hu X., Liu H., Li Q., Li T., Yang Y., Li O. (2014). Methylation patterns of the IFN-γ gene in cervical cancer tissues. Sci. Rep..

[137] Sikorski M., Bobek M., Zrubek H., Marcinkiewicz J. (2004). Dynamics of selected MHC class I and II molecule expression in the course of HPV positive CIN treatment with the use of human recombinant IFN-gamma. Acta Obstet. Gynecol. Scand..

[138] Valyi-Nagy I., Jensen P.J., Albelda S.M., Rodeck U. (1992). Cytokine-induced expression of transforming growth factor-alpha and the epidermal growth factor receptor in neonatal skin explants. J. Investig. Dermatol..

[139] Liu X. (2008). Inflammatory cytokines augments TGF-beta1-induced epithelial-mesenchymal transition in A549 cells by up-regulating TbetaR-I. Cell Motil. Cytoskelet..

[140] Hamburger A.W., Pinnamaneni G.D. (1991). Increased epidermal growth factor receptor gene expression by gamma-interferon in a human breast carcinoma cell line. Br. J. Cancer.

[141] Boente M.P., Berchuck A., Rodriguez G.C., Davidoff A., Whitaker R., Xu F.J., Marks J., Clarke-Pearson D.L., Bast R.C. (1992). The effect of interferon gamma on epidermal growth factor receptor expression in normal and malignant ovarian epithelial cells. Am. J. Obstet. Gynecol..

[142] Schmiegel W., Roeder C., Schmielau J., Rodeck U., Kalthoff H. (1993). Tumor necrosis factor alpha induces the expression of transforming growth factor alpha and the epidermal growth factor receptor in human pancreatic cancer cells. Proc. Natl. Acad. Sci. USA.

[143] Lizard G., Chignol M.C., Chardonnet Y., Schmitt D. (1996). Differences of reactivity to interferon gamma in HeLa and CaSki cells: A combined immunocytochemical and flow-cytometric study. J. Cancer Res. Clin. Oncol..

[144] Wan Y., Belt A., Wang Z., Voorhees J., Fisher G. (2001). Transmodulation of epidermal growth factor receptor mediates IL-1 beta-induced MMP-1 expression in cultured human keratinocytes. Int. J. Mol. Med..

[145] Segawa R., Shigeeda K., Hatayama T., Dong J., Mizuno N., Moriya T., Hiratsuka M., Hirasawa N. (2018). EGFR transactivation is involved in TNF-α-induced expression of thymic stromal lymphopoietin in human keratinocyte cell line. J. Dermatol. Sci..

[146] Nakayama I., Higa-Nakamine S., Uehara A., Sugahara K., Kakinohana M., Yamamoto H. (2020). Regulation of epidermal growth factor receptor expression and morphology of lung epithelial cells by interleukin-1β. J. Biochem..

[147] Haque A.S.M.R., Moriyama M., Kubota K., Ishiguro N., Sakamoto M., Chinju A., Mochizuki K., Sakamoto T., Kaneko N., Munemura R. (2019). CD206+ tumor-associated macrophages promote proliferation and invasion in oral squamous cell carcinoma via EGF production. Sci. Rep..

[148] Lee M.Y., Chou C.Y., Tang M.J., Shen M.R. (2008). Epithelial-mesenchymal transition in cervical cancer: Correlation with tumor progression, epidermal growth factor receptor overexpression, and snail up-regulation. Clin. Cancer Res..

[149] Zhen L., Fan D., Yi X., Cao X., Chen D., Wang L. (2014). Curcumin inhibits oral squamous cell carcinoma proliferation and invasion via EGFR signaling pathways. Int. J. Clin. Exp. Pathol..

[150] Kim J.W., Kim Y.T., Kim D.K., Song C.H., Lee J.W. (1996). Expression of epidermal growth factor receptor in carcinoma of the cervix. Gynecol. Oncol..

[151] Boiko I.V., Mitchell M.F., Hu W., Pandey D.K., Mathevet P., Malpica A., Hittelman W.N. (1998). Epidermal growth factor receptor expression in cervical intraepithelial neoplasia and its modulation during an alpha-difluoromethylornithine chemoprevention trial. Clin. Cancer Res..

[152] Lesur O., Brisebois M., Thibodeau A., Chagnon F., Lane D., Füllöp T. (2004). Role of IFN-gamma and IL-2 in rat lung epithelial cell migration and apoptosis after oxidant injury. Am. J. Physiol. Lung Cell Mol. Physiol..

[153] Jiang G.X., Zhong X.Y., Cui Y.F., Liu W., Tai S., Wang Z.D., Shi Y.G., Zhao S.Y., Li C.L. (2010). IL-6/STAT3/TFF3 signaling regulates human biliary epithelial cell migration and wound healing in vitro. Mol. Biol. Rep..

[154] Wang C.H., Cao G.F., Jiang Q., Yao J. (2012). TNF-α promotes human retinal pigment epithelial (RPE) cell migration by inducing matrix metallopeptidase 9 (MMP-9) expression through activation of Akt/mTORC1 signaling. Biochem. Biophys. Res. Commun..

[155] Tseng H.C., Lee I.T., Lin C.C., Chi P.L., Cheng S.E., Shih R.H., Hsiao L.D., Yang C.M. (2013). IL-1β promotes corneal epithelial cell migration by increasing MMP-9 expression through NF-κB- and AP-1-dependent pathways. PLoS ONE.

[156] Taipale J., Matikainen S., Hurme M., Keski-Oja J. (1994). Induction of transforming growth factor beta 1 and its receptor expression during myeloid leukemia cell differentiation. Cell Growth Differ..

[157] Yamauchi Y., Kohyama T., Takizawa H., Kamitani S., Desaki M., Takami K., Kawasaki S., Kato J., Nagase T. (2010). Tumor necrosis factor-alpha enhances both epithelial-mesenchymal transition and cell contraction induced in A549 human alveolar epithelial cells by transforming growth factor-beta1. Exp. Lung Res..

[158] Kamitani S., Yamauchi Y., Kawasaki S., Takami K., Takizawa H., Nagase T., Kohyama T. (2011). Simultaneous stimulation with TGF-β1 and TNF-α induces epithelial mesenchymal transition in bronchial epithelial cells. Int. Arch. Allergy Immunol..

[159] Voloshenyuk T.G., Hart A.D., Khoutorova E., Gardner J.D. (2011). TNF-α increases cardiac fibroblast lysyl oxidase expression through TGF-β and PI3Kinase signaling pathways. Biochem. Biophys. Res. Commun..

[160] Cheng K., Hao M. (2016). Metformin Inhibits TGF-β1-Induced Epithelial-to-Mesenchymal Transition via PKM2 Relative-mTOR/p70s6k Signaling Pathway in Cervical Carcinoma Cells. Int. J. Mol. Sci..

[161] Cheng K.Y., Hao M. (2017). Mammalian Target of Rapamycin (mTOR) Regulates Transforming Growth Factor-β1 (TGF-β1)-Induced Epithelial-Mesenchymal Transition via Decreased Pyruvate Kinase M2 (PKM2) Expression in Cervical Cancer Cells. Med. Sci. Monit..

[162] Kim K.K., Sheppard D., Chapman H.A. (2018). TGF-β1 Signaling and Tissue Fibrosis. Cold Spring Harb. Perspect. Biol..

[163] Sitole B.N., Mavri-Damelin D. (2018). Peroxidasin is regulated by the epithelial-mesenchymal transition master transcription factor Snai1. Gene.

[164] Tyszka-Czochara M., Lasota M., Majka M. (2018). Caffeic Acid and Metformin Inhibit Invasive Phenotype Induced by TGF-β1 in C-4I and HTB-35/SiHa human Cervical Squamous Carcinoma Cells by Acting on Different Molecular Targets. Int. J. Mol. Sci..

[165] Ji H., Liu G., Han J., Zhu F., Dong X., Li B. (2020). C-phycocyanin inhibits epithelial-to-mesenchymal transition in Caski cells. Cancer Cell Int..

[166] Stolfi C., Troncone E., Marafini I., Monteleone G. (2020). Role of TGF-Beta and Smad7 in Gut Inflammation, Fibrosis and Cancer. Biomolecules.

[167] Zhang L., Zhou F., van Dinther M., Ten Dijke P. (2016). Determining TGF-β Receptor Levels in the Cell Membrane. Methods Mol. Biol..

[168] Baritaki S., Sifakis S., Huerta-Yepez S., Neonakis I.K., Soufla G., Bonavida B., Spandidos D.A. (2007). Overexpression of VEGF and TGF-beta1 mRNA in Pap smears correlates with progression of cervical intraepithelial neoplasia to cancer: Implication of YY1 in cervical tumorigenesis and HPV infection. Int. J. Oncol..

[169] Viloria M.E., Bravo J., Carrero Y., Mosquera J.A. (2018). In situ expressions of protein 16 (p16CDKN2A) and transforming growth factor beta-1 in patients with cervical intraepithelial neoplasia and cervical cancer. Eur. J. Obstet. Gynecol. Reprod. Biol..

[170] Xu Q., Wang S., Xi L., Wu S., Chen G., Zhao Y., Wu Y., Ma D. (2006). Effects of human papillomavirus type 16 E7 protein on the growth of cervical carcinoma cells and immuno-escape through the TGF-beta1 signaling pathway. Gynecol. Oncol..

[171] Mo N., Li Z.Q., Li J., Cao Y.D. (2012). Curcumin inhibits TGF-β1-induced MMP-9 and invasion through ERK and Smad signaling in breast cancer MDA- MB-231 cells. Asian Pac. J. Cancer Prev..

[172] Huang Y., Yu T., Fu X., Chen J., Liu Y., Li C., Xia Y., Zhang Z., Li L. (2013). EGFR inhibition prevents in vitro tumor growth of salivary adenoid cystic carcinoma. BMC Cell Biol..

[173] Huang W.C., Wu S.J., Tu R.S., Lai Y.R., Liou C.J. (2015). Phloretin inhibits interleukin-1β-induced COX-2 and ICAM-1 expression through inhibition of MAPK, Akt, and NF-κB signaling in human lung epithelial cells. Food Funct..

[174] Jiménez-Garduño A.M., Mendoza-Rodríguez M.G., Urrutia-Cabrera D., Domínguez-Robles M.C., Pérez-Yépez E.A., Ayala-Sumuano J.T., Meza I. (2017). IL-1β induced methylation of the estrogen receptor ERα gene correlates with EMT and chemoresistance in breast cancer cells. Biochem Biophys Res Commun.

[175] Nishikai-Yan Shen T., Kanazawa S., Kado M., Okada K., Luo L., Hayashi A., Mizuno H., Tanaka R. (2017). Interleukin-6 stimulates Akt and p38 MAPK phosphorylation and fibroblast migration in non-diabetic but not diabetic mice. PLoS ONE.

[176] Yang D., Xiao C.X., Su Z.H., Huang M.W., Qin M., Wu W.J., Jia W.W., Zhu Y.Z., Hu J.F., Liu X.H. (2017). (-)-7(S)-hydroxymatairesinol protects against tumor necrosis factor-α-mediated inflammation response in endothelial cells by blocking the MAPK/NF-κB and activating Nrf2/HO-1. Phytomedicine.

[177] Hamidi A., Song J., Thakur N., Itoh S., Marcusson A., Bergh A., Heldin C.H., Landström M. (2017). TGF-β promotes PI3K-AKT signaling and prostate cancer cell migration through the TRAF6-mediated ubiquitylation of p85α. Sci. Signal..

[178] Rodríguez-García A., Samsó P., Fontova P., Simon-Molas H., Manzano A., Castaño E., Rosa J.L., Martinez-Outshoorn U., Ventura F., Navarro-Sabaté À. (2017). TGF-β1 targets Smad, p38 MAPK, and PI3K/Akt signaling pathways to induce PFKFB3 gene expression and glycolysis in glioblastoma cells. FEBS J..

[179] Wang Y., Wan D., Zhou R., Zhong W., Lu S., Chai Y. (2017). Geraniin inhibits migration and invasion of human osteosarcoma cancer cells through regulation of PI3K/Akt and ERK1/2 signaling pathways. Anticancer Drugs.

[180] Bai T., Liu F., Zou F., Zhao G., Jiang Y., Liu L., Shi J., Hao D., Zhang Q., Zheng T. (2017). Epidermal Growth Factor Induces Proliferation of Hair Follicle-Derived Mesenchymal Stem Cells Through Epidermal Growth Factor Receptor-Mediated Activation of ERK and AKT Signaling Pathways Associated with Upregulation of Cyclin D1 and Downregulation of p16. Stem Cells Dev..

[181] Zibara K., Zeidan A., Bjeije H., Kassem N., Badran B., El-Zein N. (2017). ROS mediates interferon gamma induced phosphorylation of Src, through the Raf/ERK pathway, in MCF-7 human breast cancer cell line. J. Cell Commun. Signal..

[182] Joseph J.P., Harishankar M.K., Pillai A.A., Devi A. (2018). Hypoxia induced EMT: A review on the mechanism of tumor progression and metastasis in OSCC. Oral Oncol..

[183] Tripathy J., Tripathy A., Thangaraju M., Suar M., Elangovan S. (2018). α-Lipoic acid inhibits the migration and invasion of breast cancer cells through inhibition of TGFβ signaling. Life Sci..

[184] Morales-Garcia V., Contreras-Paredes A., Martinez-Abundis E., Gomez-Crisostomo N.P., Lizano M., Hernandez-Landero F., de la Cruz-Hernandez E. (2020). The high-risk HPV E6 proteins modify the activity of the eIF4E protein via the MEK/ERK and AKT/PKB pathways. FEBS Open Bio.

[185] Jin J., Zhang Z., Zhang S., Chen X., Chen Z., Hu P., Wang J., Xie C. (2018). Fatty acid binding protein 4 promotes epithelial-mesenchymal transition in cervical squamous cell carcinoma through AKT/GSK3β/Snail signaling pathway. Mol. Cell. Endocrinol..

[186] Hernández-Padilla L., Reyes de la Cruz H., Campos-García J. (2020). Antiproliferative effect of bacterial cyclodipeptides in the HeLa line of human cervical cancer reveals multiple protein kinase targeting, including mTORC1/C2 complex inhibition in a TSC1/2-dependent manner. Apoptosis.

[187] Wang D., Li Q., Li K., Xiao P., Yin R. (2015). Twist-related protein 1-mediated regulation of mesenchymal change contributes to the migration and invasion of cervical cancer cells. Oncol. Lett..

[188] Srinivas K.P., Viji R., Dan V.M., Sajitha I.S., Prakash R., Rahul P.V., Santhoshkumar T.R., Lakshmi S., Pillai M.R. (2016). DEPTOR promotes survival of cervical squamous cell carcinoma cells and its silencing induces apoptosis through downregulating PI3K/AKT and by up-regulating p38 MAP kinase. Oncotarget.

[189] Liu C., Ding L., Bai L., Chen X., Kang H., Hou L., Wang J. (2017). Folate receptor alpha is associated with cervical carcinogenesis and regulates cervical cancer cells growth by activating ERK1/2/c-Fos/c-Jun. Biochem. Biophys. Res. Commun..

[190] Saxena K., Jolly M.K., Balamurugan K. (2020). Hypoxia, partial EMT and collective migration: Emerging culprits in metastasis. Transl. Oncol..

[191] Ghatak D., Das Ghosh D., Roychoudhury S. (2021). Cancer Stemness: p53 at the Wheel. Front. Oncol..

[192] Viallard C., Larrivée B. (2017). Tumor angiogenesis and vascular normalization: Alternative thera-peutic targets. Angiogenesis.

[193] Pezzuto A., Carico E. (2018). Role of HIF-1 in Cancer Progression: Novel Insights. A Review. Curr. Mol. Med..

[194] Acs G., Zhang P.J., McGrath C.M., Acs P., McBroom J., Mohyeldin A., Liu S., Lu H., Verma A. (2003). Hypoxia-inducible erythropoietin signaling in squamous dysplasia and squamous cell carcinoma of the uterine cervix and its potential role in cervical carcinogenesis and tumor progression. Am. J. Pathol..

[195] Sartori-Cintra A.R., Mara C.S., Argolo D.L., Coimbra I.B. (2012). Regulation of hypoxia-inducible factor-1α (HIF-1α) expression by interleukin-1β (IL-1 β), insulin-like growth factors I (IGF-I) and II (IGF-II) in human osteoarthritic chondrocytes. Clinics.

[196] Zhang H.X., Yang J.J., Zhang S.A., Zhang S.M., Wang J.X., Xu Z.Y., Lin R.Y. (2018). HIF-1α promotes inflammatory response of chronic obstructive pulmonary disease by activating EGFR/PI3K/AKT pathway. Eur. Rev. Med. Pharmacol. Sci..

[197] Gao X., Li Y., Wang H., Li C., Ding J. (2017). Inhibition of HIF-1α decreases expression of pro-inflammatory IL-6 and TNF-α in diabetic retinopathy. Acta Ophthalmol..

[198] Ogryzko N.V., Lewis A., Wilson H.L., Meijer A.H., Renshaw S.A., Elks P.M. (2019). Hif-1α-Induced Expression of Il-1β Protects against Mycobacterial Infection in Zebrafish. J. Immunol..

[199] Warbrick I., Rabkin S.W. (2019). Hypoxia-inducible factor 1-alpha (HIF-1α) as a factor mediating the relationship between obesity and heart failure with preserved ejection fraction. Obes. Rev..

[200] Kim K.W., Lee S.J., Kim J.C. (2017). TNF-α upregulates HIF-1α expression in pterygium fibroblasts and enhances their susceptibility to VEGF independent of hypoxia. Exp. Eye Res..

[201] Xu S., Yu C., Ma X., Li Y., Shen Y., Chen Y., Huang S., Zhang T., Deng W., Wang Y. (2021). IL-6 promotes nuclear translocation of HIF-1α to aggravate chemoresistance of ovarian cancer cells. Eur. J. Pharmacol..

[202] Nakamura M., Bodily J.M., Beglin M., Kyo S., Inoue M., Laimins L.A. (2009). Hypoxia-specific stabilization of HIF-1alpha by human papillomaviruses. Virology.

[203] Rodolico V., Arancio W., Amato M.C., Aragona F., Cappello F., Di Fede O., Pannone G., Campisi G. (2011). Hypoxia inducible factor-1 alpha expression is increased in infected positive HPV16 DNA oral squamous cell carcinoma and positively associated with HPV16 E7 oncoprotein. Infect. Agent Cancer.

[204] Fan R., Hou W.J., Zhao Y.J., Liu S.L., Qiu X.S., Wang E.H., Wu G.P. (2016). Overexpression of HPV16 E6/E7 mediated HIF-1α upregulation of GLUT1 expression in lung cancer cells. Tumour Biol..

[205] Fujiwaki R., Hata K., Iida K., Maede Y., Miyazaki K. (2000). Vascular endothelial growth factor expression in progression of cervical cancer: Correlation with thymidine phosphorylase expression, angiogenesis, tumor cell proliferation, and apoptosis. Anticancer Res..

[206] Triratanachat S., Niruthisard S., Trivijitsilp P., Tresukosol D., Jarurak N. (2006). Angiogenesis in cervical intraepithelial neoplasia and early-staged uterine cervical squamous cell carcinoma: Clinical significance. Int. J. Gynecol. Cancer.

[207] Kim N.S., Kang Y.J., Jo J.O., Kim H.Y., Oh Y.R., Kim Y.O., Jung M.H., Ock M.S., Cha H.J. (2011). Elevated expression of thymosin β4, vascular endothelial growth factor (VEGF), and hypoxia inducible factor (HIF)-1α in early-stage cervical cancers. Pathol. Oncol. Res..

[208] Durand R.E., Aquino-Parsons C. (2006). The fate of hypoxic (pimonidazole-labelled) cells in human cervix tumours undergoing chemo-radiotherapy. Radiother. Oncol..

[209] Sundfør K., Lyng H., Rofstad E.K. (1998). Tumour hypoxia and vascular density as predictors of metastasis in squamous cell carcinoma of the uterine cervix. Br. J. Cancer.

[210] Höckel M., Schlenger K., Höckel S., Vaupel P. (1999). Hypoxic cervical cancers with low apoptotic index are highly aggressive. Cancer Res..

[211] Chen H., Chan D.C. (2017). Mitochondrial Dynamics in Regulating the Unique Phenotypes of Cancer and Stem Cells. Cell Metab..

[212] Sachdeva M., Zhu S., Wu F., Wu H., Walia V., Kumar S., Elble R., Watabe K., Mo Y.Y. (2009). p53 represses c-Myc through induction of the tumor suppressor miR-145. Proc. Natl. Acad. Sci. USA.

[213] Jain A.K., Allton K., Iacovino M., Mahen E., Milczarek R.J., Zwaka T.P., Kyba M., Barton M.C. (2012). p53 regulates cell cycle and microRNAs to promote differentiation of human embryonic stem cells. PLoS Biol..

[214] Lu Y., Zhang K., Li C., Yao Y., Tao D., Liu Y., Zhang S., Ma Y. (2012). Piwil2 suppresses p53 by inducing phosphorylation of signal transducer and activator of transcription 3 in tumor cells. PLoS ONE.

[215] Li H., Rokavec M., Jiang L., Horst D., Hermeking H. (2017). Antagonistic Effects of p53 and HIF1A on microRNA-34a Regulation of PPP1R11 and STAT3 and Hypoxia-induced Epithelial to Mesenchymal Transition in Colorectal Cancer Cells. Gastroenterology.

[216] Liu X., Fan D. (2015). The epithelial-mesenchymal transition and cancer stem cells: Functional and mechanistic links. Curr. Pharm. Des..

[217] Xia P., Xu X.Y. (2017). Epithelial-mesenchymal transition and gastric cancer stem cell. Tumour Biol..

[218] Du B., Shim J.S. (2016). Targeting Epithelial-Mesenchymal Transition (EMT) to Overcome Drug Resistance in Cancer. Molecules.

[219] Shibue T., Weinberg R.A. (2017). EMT, CSCs, and drug resistance: The mechanistic link and clinical implications. Nat. Rev. Clin. Oncol..

[220] Li S.W., Wu X.L., Dong C.L., Xie X.Y., Wu J.F., Zhang X. (2015). The differential expression of OCT4 isoforms in cervical carcinoma. PLoS ONE.

[221] Su P.H., Hsu Y.W., Huang R.L., Chen L.Y., Chao T.K., Liao C.C., Chen C.W., Wu T.I., Mao S.P., Balch C. (2019). TET1 promotes 5hmC-dependent stemness, and inhibits a 5hmC-independent epithelial-mesenchymal transition, in cervical precancerous lesions. Cancer Lett..

[222] Zhang J., Chen X., Bian L., Wang Y., Liu H. (2019). CD44+/CD24+-Expressing Cervical Cancer Cells and Radioresistant Cervical Cancer Cells Exhibit Cancer Stem Cell Characteristics. Gynecol. Obstet. Investig..

[223] De Brux J. (1982). Natural history of the cervical precancerous lesions and their evolutions. Eur. J. Gynaecol. Oncol..

[224] Organista-Nava J., Gómez-Gómez Y., Gariglio P. (2014). Embryonic stem cell-specific signature in cervical cancer. Tumour Biol..

[225] Xu J., Wang H., Wang H., Chen Q., Zhang L., Song C., Zhou Q., Hong Y. (2019). The inhibition of miR-126 in cell migration and invasion of cervical cancer through regulating ZEB1. Hereditas.

[226] Tian W., Zhang W., Zhang Y., Zhu T., Hua Y., Li H., Zhang Q., Xia M. (2020). FABP4 promotes invasion and metastasis of colon cancer by regulating fatty acid transport. Cancer Cell Int..

[227] Mun J.G., Han Y.H., Jeon H.D., Yoon D.H., Lee Y.G., Hong S.H., Kee J.Y. (2021). Inhibitory Effect of Gallotannin on Lung Metastasis of Metastatic Colorectal Cancer Cells by Inducing Apoptosis, Cell Cycle Arrest and Autophagy. Am. J. Chin. Med..

[228] Zheng L., Li N., Guo F., Jian X.C., Jiang C.H., Yin P., Min A.J., Huang L. (2015). Twist-related protein 1 enhances oral tongue squamous cell carcinoma cell invasion through β-catenin signaling. Mol. Med. Rep..

[229] Kashyap T., Nath N., Mishra P., Jha A., Nagini S., Mishra R. (2020). Pluripotency transcription factor Nanog and its association with overall oral squamous cell carcinoma progression, cisplatin-resistance, invasion and stemness acquisition. Head Neck.

[230] Zheng J.H., Jiao S.J., Na L., Zheng S.Q., Ma Z.H., Wang S.W., Aili A., Hasim A. (2017). Defective expression of polarity protein Par3 promotes cervical tumorigenesis and metastasis. Eur. J. Gynaecol. Oncol..

[231] Lu K., Dong J.L., Fan W.J. (2018). Twist1/2 activates MMP2 expression via binding to its promoter in colorectal cancer. Eur. Rev. Med. Pharmacol. Sci..

[232] Zhang Y., Wang X. (2020). Targeting the Wnt/β-catenin signaling pathway in cancer. J. Hematol. Oncol..

[233] Chang B., Kim J., Jeong D., Jeong Y., Jeon S., Jung S.I., Yang Y., Kim K.I., Lim J.S., Kim C. (2012). Klotho inhibits the capacity of cell migration and invasion in cervical cancer. Oncol. Rep..

[234] Zhou Y., Huang Y., Cao X., Xu J., Zhang L., Wang J., Huang L., Huang S., Yuan L., Jia W. (2016). WNT2 Promotes Cervical Carcinoma Metastasis and Induction of Epithelial-Mesenchymal Transition. PLoS ONE.

[235] Müller T., Bain G., Wang X., Papkoff J. (2002). Regulation of epithelial cell migration and tumor formation by beta-catenin signaling. Exp. Cell Res..

[236] Kim S.E., Choi K.Y. (2007). EGF receptor is involved in WNT3a-mediated proliferation and motility of NIH3T3 cells via ERK pathway activation. Cell Signal..

[237] Shi I., Hashemi Sadraei N., Duan Z.H., Shi T. (2011). Aberrant signaling pathways in squamous cell lung carcinoma. Cancer Inform..

[238] Zhou S. (2011). TGF-β regulates β-catenin signaling and osteoblast differentiation in human mesenchymal stem cells. J. Cell Biochem..

[239] Landman E.B., Miclea R.L., van Blitterswijk C.A., Karperien M. (2013). Small molecule inhibitors of WNT/β-catenin signaling block IL-1β- and TNFα-induced cartilage degradation. Arthritis Res. Ther..

[240] Zhang W., Zhang H., Wang N., Zhao C., Zhang H., Deng F., Wu N., He Y., Chen X., Zhang J. (2013). Modulation of β-catenin signaling by the inhibitors of MAP kinase, tyrosine kinase, and PI3-kinase pathways. Int. J. Med. Sci..

[241] Zhang H., Nan W., Wang S., Zhang T., Si H., Yang F., Li G. (2016). Epidermal Growth Factor Promotes Proliferation and Migration of Follicular Outer Root Sheath Cells via Wnt/β-Catenin Signaling. Cell Physiol. Biochem..

[242] Ratz L., Laible M., Kacprzyk L.A., Wittig-Blaich S.M., Tolstov Y., Duensing S., Altevogt P., Klauck S.M., Sültmann H. (2017). TMPRSS2/ERG gene fusion variants induce TGF-β signaling and epithelial to mesenchymal transition in human prostate cancer cells. Oncotarget.

[243] Gao S., Hu J., Wu X., Liang Z. (2018). PMA treated THP-1-derived-IL-6 promotes EMT of SW48 through STAT3/ERK-dependent activation of Wnt/β-catenin signaling pathway. Biomed. Pharm..

[244] Hu Y., Chen W., Wu L., Jiang L., Liang N., Tan L., Liang M., Tang N. (2019). TGF-β1 Restores Hippocampal Synaptic Plasticity and Memory in Alzheimer Model via the PI3K/Akt/Wnt/β-Catenin Signaling Pathway. J. Mol. Neurosci..

[245] Lee M.S., Lee J., Kim Y.M., Lee H. (2019). The metastasis suppressor CD82/KAI1 represses the TGF-β 1 and Wnt signalings inducing epithelial-to-mesenchymal transition linked to invasiveness of prostate cancer cells. Prostate.

[246] Bas E., Anwar M.R., Van De Water T.R. (2020). TGF β-1 and WNT Signaling Pathways Collaboration Associated with Cochlear Implantation Trauma-Induced Fibrosis. Anat. Rec..

[247] Du L., Lee J.H., Jiang H., Wang C., Wang S., Zheng Z., Shao F., Xu D., Xia Y., Li J. (2020). β-Catenin induces transcriptional expression of PD-L1 to promote glioblastoma immune evasion. J. Exp. Med..

[248] Han J., Shen X., Zhang Y., Wang S., Zhou L. (2020). Astragaloside IV suppresses transforming growth factor-β1-induced epithelial-mesenchymal transition through inhibition of Wnt/β-catenin pathway in glioma U251 cells. Biosci. Biotechnol. Biochem..

[249] Seomun Y., Kim J.T., Joo C.K. (2008). MMP-14 mediated MMP-9 expression is involved in TGF-beta1-induced keratinocyte migration. J. Cell Biochem..

[250] Sinpitaksakul S.N., Pimkhaokham A., Sanchavanakit N., Pavasant P. (2008). TGF-beta1 induced MMP-9 expression in HNSCC cell lines via Smad/MLCK pathway. Biochem. Biophys. Res. Commun..

[251] Chang C.C., Ling X.H., Hsu H.F., Wu J.M., Wang C.P., Yang J.F., Fang L.W., Houng J.Y. (2016). Siegesbeckia orientalis Extract Inhibits TGFβ1-Induced Migration and Invasion of Endometrial Cancer Cells. Molecules.

[252] Huang Z., Li S., Fan W., Ma Q. (2017). Transforming growth factor β1 promotes invasion of human JEG-3 trophoblast cells via TGF-β/Smad3 signaling pathway. Oncotarget.

[253] Xie F., Jin K., Shao L., Fan Y., Tu Y., Li Y., Yang B., van Dam H., Ten Dijke P., Weng H. (2017). FAF1 phosphorylation by AKT accumulates TGF-β type II receptor and drives breast cancer metastasis. Nat. Commun..

[254] Pramanik K.K., Nagini S., Singh A.K., Mishra P., Kashyap T., Nath N., Alam M., Rana A., Mishra R. (2018). Glycogen synthase kinase-3β mediated regulation of matrix metalloproteinase-9 and its involvement in oral squamous cell carcinoma progression and invasion. Cell. Oncol..

[255] Drews C.M., Case S., Vande Pol S.B. (2019). E6 proteins from high-risk HPV, low-risk HPV, and animal papillomaviruses activate the Wnt/β-catenin pathway through E6AP-dependent degradation of NHERF1. PLoS Pathog..

[256] Li B., Guo X., Li N., Chen Q., Shen J., Huang X., Huang G., Wang F. (2020). WNT1, a target of miR-34a, promotes cervical squamous cell carcinoma proliferation and invasion by induction of an E-P cadherin switch via the WNT/β-catenin pathway. Cell. Oncol..

[257] Choi J.Y., Jang Y.S., Min S.Y., Song J.Y. (2011). Overexpression of MMP-9 and HIF-1α in Breast Cancer Cells under Hypoxic Conditions. J. Breast Cancer.

[258] Tinganelli W., Durante M. (2020). Tumor Hypoxia and Circulating Tumor Cells. Int. J. Mol. Sci..

[259] Jiang J., Li X., Yin X., Zhang J., Shi B. (2019). Association of low expression of E-cadherin and β-catenin with the progression of early stage human squamous cervical cancer. Oncol. Lett..

[260] Zhou S., Yang J., Wang M., Zheng D., Liu Y. (2020). Endoplasmic reticulum stress regulates epithelial mesenchymal transition in human lens epithelial cells. Mol. Med. Rep..

[261] Eddy R.J., Weidmann M.D., Sharma V.P., Condeelis J.S. (2017). Tumor Cell Invadopodia: Invasive Protrusions that Orchestrate Metastasis. Trends Cell Biol..

[262] Stenman J., Lintula S., Hotakainen K., Vartiainen J., Lehväslaiho H., Stenman U.H. (1997). Detection of squamous-cell carcinoma antigen-expressing tumour cells in blood by reverse transcriptase-polymerase chain reaction in cancer of the uterine cervix. Int. J. Cancer.

[263] Wei X.Q., Ma Y., Chen Y., Liu X., Zhao M., Zhou L.W. (2018). Laparoscopic surgery for early cervical squamous cell carcinoma and its effect on the micrometastasis of cancer cells. Medicine.

[264] Wen Y.F., Cheng T.T., Chen X.L., Huang W.J., Peng H.H., Zhou T.C., Lin X.D., Zeng L.S. (2018). Elevated circulating tumor cells and squamous cell carcinoma antigen levels predict poor survival for patients with locally advanced cervical cancer treated with radiotherapy. PLoS ONE.

[265] Braunholz D., Saki M., Niehr F., Öztürk M., Borràs Puértolas B., Konschak R., Budach V., Tinhofer I. (2016). Spheroid Culture of Head and Neck Cancer Cells Reveals an Important Role of EGFR Signalling in Anchorage Independent Survival. PLoS ONE.

[266] Micalizzi D.S., Haber D.A., Maheswaran S. (2017). Cancer metastasis through the prism of epithelial-to-mesenchymal transition in circulating tumor cells. Mol. Oncol..

[267] Rudzinski J.K., Govindasamy N.P., Asgari A., Saito M.S., Lewis J.D., Jurasz P. (2021). Preferential interaction of platelets with prostate cancer cells with stem cell markers. Thromb. Res..

[268] St Hill C.A. (2011). Interactions between endothelial selectins and cancer cells regulate metastasis. Front. Biosci..

[269] Fischer V., Wong M., Li F.Q., Takemaru K.I. (2017). Chibby1 knockdown promotes mesenchymal-to-epithelial transition-like changes. Cell Cycle.

[270] Dellas K., Bache M., Pigorsch S.U., Taubert H., Kappler M., Holzapfel D., Zorn E., Holzhausen H.J., Haensgen G. (2008). Prognostic impact of HIF-1alpha expression in patients with definitive radiotherapy for cervical cancer. Strahlenther. Onkol..

[271] Hou T., Zhang W., Tong C., Kazobinka G., Huang X., Huang Y., Zhang Y. (2015). Putative stem cell markers in cervical squamous cell carcinoma are correlated with poor clinical outcome. BMC Cancer.

[272] Shen L., Huang X., Xie X., Su J., Yuan J., Chen X. (2014). High Expression of SOX2 and OCT4 Indicates Radiation Resistance and an Independent Negative Prognosis in Cervical Squamous Cell Carcinoma. J. Histochem. Cytochem..

[273] Zamulaeva I.A., Selivanova E.I., Kiseleva V.I., Matchuk O.N., Krikunova L.I., Mkrtchyan L.S., Kaprin A.D. (2020). Correlation of Radiation Response of Cervical Cancer Stem Cells with Their Initial Number before Treatment and Molecular Genetic Features of Papillomavirus Infection. Bull. Exp. Biol. Med..

[274] Jarosz-Biej M., Smolarczyk R., Cichoń T., Kułach N. (2019). Tumor Microenvironment as A “Game Changer” in Cancer Radiotherapy. Int. J. Mol. Sci..

[275] Joura E.A., Ulied A., Vandermeulen C., Rua Figueroa M., Seppä I., Hernandez Aguado J.J., Ahonen A., Reich O., Virta M., Perino A. (2021). Immunogenicity and safety of a nine-valent human papillomavirus vaccine in women 27–45 years of age compared to women 16–26 years of age: An open-label phase 3 study. Vaccine.

[276] Ge Y., Liu Y., Cheng Y., Liu Y. (2021). Predictors of recurrence in patients with high-grade cervical intraepithelial neoplasia after cervical conization. Medicine.

[277] Hong K.O., Kim J.H., Hong J.S., Yoon H.J., Lee J.I., Hong S.P., Hong S.D. (2009). Inhibition of Akt activity induces the mesenchymal-to-epithelial reverting transition with restoring E-cadherin expression in KB and KOSCC-25B oral squamous cell carcinoma cells. J. Exp. Clin. Cancer Res..

[278] De Amicis F., Perri A., Vizza D., Russo A., Panno M.L., Bonofiglio D., Giordano C., Mauro L., Aquila S., Tramontano D. (2013). Epigallocatechin gallate inhibits growth and epithelial-to-mesenchymal transition in human thyroid carcinoma cell lines. J. Cell. Physiol..

[279] Deng B., Zhang S., Miao Y., Zhang Y., Wen F., Guo K. (2015). Down-regulation of Frizzled-7 expression inhibits migration, invasion, and epithelial-mesenchymal transition of cervical cancer cell lines. Med. Oncol..

[280] He W., Tan R., Dai C., Li Y., Wang D., Hao S., Kahn M., Liu Y. (2010). Plasminogen activator inhibitor-1 is a transcriptional target of the canonical pathway of Wnt/beta-catenin signaling. J. Biol. Chem..

[281] Yang X., Li S., Li W., Chen J., Xiao X., Wang Y., Yan G., Chen L. (2013). Inactivation of lysyl oxidase by β-aminopropionitrile inhibits hypoxia-induced invasion and migration of cervical cancer cells. Oncol. Rep..

